# Asymmetric two-photon response of an incoherently driven quantum emitter

**DOI:** 10.1038/s41467-026-72706-z

**Published:** 2026-05-09

**Authors:** Lennart Jehle, Lena M. Hansen, Patrik I. Sund, Thomas W. Sandø, Raphael Joos, Michael Jetter, Simone L. Portalupi, Mathieu Bozzio, Peter Michler, Philip Walther

**Affiliations:** 1 University of Vienna, Faculty of Physics, Vienna Center for Quantum Science and Technology (VCQ), Vienna, Austria; 2 Christian Doppler Laboratory for Photonic Quantum Computer, University of Vienna, Vienna, Austria; 3 Institut für Halbleiteroptik und Funktionelle Grenzflächen, Center for Integrated Quantum Science and Technology (IQST) and SCoPE, University of Stuttgart, Stuttgart, Germany; 4 Institute for Quantum Optics and Quantum Information (IQOQI) Vienna, Austrian Academy of Sciences, Vienna, Austria

**Keywords:** Single photons and quantum effects, Quantum information

## Abstract

Quantum emitters promise to emit exactly one photon when pumped by a laser pulse. However, even in ideal systems, re-excitation during the laser pulse duration causes the consecutive emission of two photons, thus limiting the single-photon purity. Although the probability and characteristics of re-excitation are largely determined by the excitation scheme, until now, only resonant driving has been studied. Here, we demonstrate qualitative differences for phonon-assisted excitation and resolve the distinct temporal and spectral profile of each of the photons. These uncover correlations between the emission time and wavelength, resulting in an asymmetric two-photon spectrum. On a fundamental level, we show that this spectrum provides direct access to the Rabi frequency of an incoherently driven quantum dot. On the application side, we leverage the asymmetry to selectively suppress multiphoton noise, ensuring high single-photon purity regardless of the laser pulse length and thus enhancing implementations across quantum cryptography and quantum computing.

## Abstract (web summary)

Re-excitation, whereby a single excitation pulse leads to the emission of multiple photons, limits the single-photon purity of quantum emitters. Here, by examining the re-excitation dynamics of a quantum dot under incoherent excitation, the authors resolve spectro-temporal correlations in the emission that can be used to suppress multiphoton noise and boost performance of a single-photon source.

## Introduction

Studying the rich physics of light–matter interaction not only provides valuable insights into fundamental quantum processes but also inspires the design of applications that benefit from these processes. Among the variety of quantum emitters that allow such studies, quantum dots (QDs) have proven to be a powerful platform for foundational experiments in quantum optics^1–4^ and applications in both quantum communication^5–9^ and computing^10–13^, thanks to simultaneously high single-photon efficiencies^14^, low multiphoton contribution^15,16^ and highly indistinguishable emission^17,18^. A large part of the success stems from the high level of control gained by embedding these artificial atoms into a semiconductor host structure, which allows the integration of photonic cavities to improve collection efficiencies and provide Purcell enhancement, as well as electrical diodes^19,20^ to stabilize the charge environment and tune the emission wavelength. However, the benefits of stable semiconductor integration also come with a coupling to the surrounding environment—most notably to charge noise, spin reservoirs, and the phonon bath. When unmanaged, such interactions can impair the properties of the emitter, as evidenced by damped Rabi oscillations^21^, diminished photon indistinguishability^22,23^, or reduced spin coherence times^24,25^. Yet, when understood and used to our advantage, these very interactions also offer opportunities, including spin refocussing^26^, control over the charge state^20^, or phonon-mediated schemes to incoherently excite the quantum emitter^27–29^.

In recent years, longitudinal acoustic (LA) phonon-assisted pumping^30^ has attracted significant interest for driving QDs^31–34^, defects in hBN^35^ or delocalized excitions in transition metal dichalcogenides^36^. The spectral width of the exciton–phonon interaction ensures an intrinsic robustness against laser instabilities^31^ and enables straightforward suppression of laser background via frequency filtering. The latter renders LA excitation ideal for generating polarization-entangled linear cluster states from QDs^32,37^, while applications in quantum communication additionally benefit from the phase randomization required in many cryptographic protocols and provided by the coupling to the phonon bath^33^.

As a result of continuous fabrication improvements, fundamental challenges—rather than technical imperfections—are now beginning to limit the achievable performance^38,39^. Among these, the impact of re-excitation, where a single laser pulse may induce the emission of multiple photons^40^, is particularly severe as it compromises the single-photon purity of the source. In resonantly driven two-level systems, the re-excitation probability scales proportionally to the laser pulse length and the radiative decay rate^16^. However, except for initial theoretical work that focussed on adiabatic undressing^41^, the phenomenon is largely unexplored for phonon-assisted pumping. At the same time, a comprehensive understanding of the re-excitation dynamics is becoming increasingly important as recent cavity designs yield Purcell factors exceeding 40 for photonic crystals^42^ and 25 for circular Bragg gratings^43^, leading to exciton decay times as short as 23 ps. While the accelerated decay improves the photon indistinguishability and supports faster clock rates, it also increases the multiphoton emission caused by re-excitation.

Here, we present experimental results exploring both the temporal and spectral signatures of the re-excitation process of a QD under LA phonon-assisted pumping. We individually resolve the time and frequency modes of the first and second emitted photon and reveal significant differences compared to the resonant counterpart. Importantly, we observe a spectral shift for the first photon that is unique to phonon-assisted driving and can be interpreted as dynamic Stark tuning caused by the excitation laser. The shift allows us to assess the Rabi frequency of an incoherently driven emitter. Moreover, we demonstrate how these Stark-induced time-frequency correlations can be exploited to limit the multiphoton contribution irrespective of the laser pulse length only using spectral filtering.

## Results

### Re-excitation under phonon-assisted driving

**Fig. 1:**
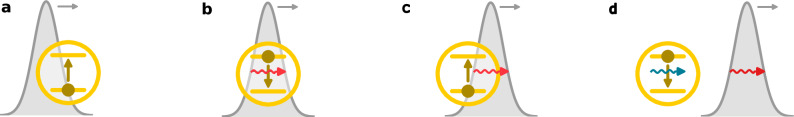
Schematic time series of a re-excitation event. Modeling the QD as a two-level system, the re-excitation process from a single laser pulse is described using a representative quantum trajectory, broken down into four distinct events. **a** Early during the interaction with the laser pulse, the QD is excited and **b** decays shortly after, emitting a 1^st^ single photon (red arrow). **c** Towards the end of the interaction with the laser pulse, the QD is excited a second time. **d** The laser pulse and the 1^st^ photon have passed, when the QD spontaneously decays again and emits the 2^nd^ photon (blue arrow). Thus, the 1^st^ and 2^nd^ photon are emitted consecutively in a strict time-ordered manner.

The re-excitation process is conceptually explained in Fig. 1 for one exemplary quantum trajectory. Independent of the pumping scheme, when driving the QD with a short laser pulse, there is a chance that it is excited early during the interaction (a) and quickly decays by emitting a 1^st^ photon (b) such that the QD can be excited again by the same pulse (c) and emit a 2^nd^ photon at a later time (d). The causality of the process requires that whenever two photons were created from one laser pulse, the 1^st^ photon must have been emitted while the pulse was still interacting with the QD (by referring to the 1^st^ or 2^nd^ photon, we imply that indeed two photons were detected). Following this reasoning, temporal filtering has been proposed to restore a high single-photon purity^44,45^ but is limited to post-selection, because the fast time scales are infeasible for current electro-optic modulators.

To understand re-excitation in the phonon-assisted scheme, we briefly recall the dressed-state picture, where the QD is treated as a two-level system with transition frequency *ω*_0_, and the laser frequency *ω*_L_ is detuned by *δω*_L_ = *ω*_L_ − *ω*_0_ (for more details, see Supplementary Note 1). Since the excitation using a negatively detuned laser would require the absorption of a phonon (which is strongly suppressed at cryogenic temperatures), we limit ourselves to *δω*_L_ > 0. The interaction of the QD with the external laser field is captured by a light–matter Hamiltonian in the rotating frame, which gives rise to a new set of eigenstates, the so-called dressed states $| \alpha,N\rangle$ and $| \beta,N\rangle$, where *N* denotes the number of laser photons^46^. At the beginning of the pulse, the ground state coincides with $| \alpha,N\rangle$ but as the laser field increases, it admixes an excitonic (ground state) component to $| \alpha,N\rangle$ ($| \beta,N\rangle$), and thereby activates phonon interactions between the dressed states^47^. When the level splitting of the dressed states—determined by the effective Rabi frequency ${\Omega }_{{\mbox{eff}}}(t)=\sqrt{{\Omega }^{2}(t)+\delta {\omega }_{{\mbox{L}}}^{2}}$—is close to the energy of maximal spectral phonon density *J*(*ω*), $| \beta,N\rangle$ can be efficiently populated through phonon emission^30^. As the laser pulse exits the system, $| \beta,N\rangle$ is adiabatically transformed back to the exciton state, which completes population inversion. Recombination at a later time then produces a single photon at *ω*_0_^47^. However, if a photon is emitted while the laser pulse is still present, we must consider the transition $| \beta,N\rangle \to | \alpha,N-1\rangle$ in the dressed-state picture.

**Fig. 2:**
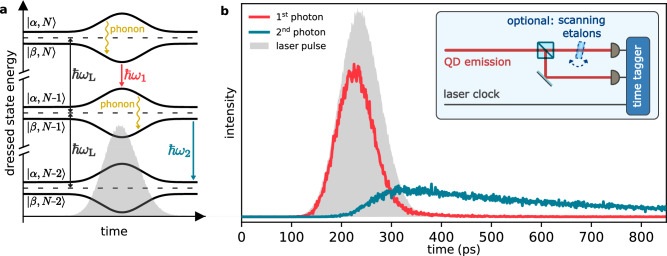
Re-excitation under phonon-assisted driving. **a** For one exemplary quantum trajectory, the re-excitation process under LA driving is schematically depicted in the energy diagram of the dynamical dressed states reduced to three manifolds of the laser field. The laser pulse is shown in gray. The emission of a first phonon sets the system into the lower dressed state $| \beta,N\rangle$ and, shortly after, but when the system is still dressed, a 1^st^ photon is emitted at *ω*_1_ by decaying into $| \alpha,N-1\rangle$. Thereafter, another phonon emission brings the system into the lower dressed state of this manifold ($| \beta,N-1\rangle$), which is adiabatically transformed into the exciton state once the laser pulse exits. The emission of a 2^nd^ photon at *ω*_2_ completes the process. The phonon coupling efficiency is governed by the phonon spectral density evaluated at the instantaneous Rabi frequency^64^. **b** Time-histogram of the 1^st^ and 2^nd^ photon showing the different temporal shapes. The laser pulse in gray is displayed for time reference. Inset: schematic detection setup in HBT configuration, additionally equipped with the electronic laser clock for advanced correlations. For more details, see Supplementary Note 4. The scanning etalons indicated by dashed lines are used only for the spectrally resolved measurements.

The complete LA re-excitation process is schematically outlined in Fig. 2a and we note two major differences compared to previously studied resonant systems. First, the excitation requires not only the absorption of a laser photon but also the emission of a phonon, which (depending on the phonon decay rate) might delay the excitation and thereby lower the re-excitation probability. Second, the eigenenergies of $| \alpha \rangle$ and $| \beta \rangle$ change antisymmetrically with the instantaneous laser field strength such that the transition frequency inherits the time dependence of the driving pulse

1 $${\omega }_{{\mbox{QD}}}(t)={\omega }_{{\mbox{L}}}-{\Omega }_{{{\mathrm{eff}}}}(t)\,.$$

This time-varying frequency shift is reminiscent of the dynamic Stark effect^48^, where a laser pulse alters the energy structure of a few-level system. However, whereas in previous works the Stark shift was purposefully induced using a secondary pump laser^48–50^, in the LA scheme the excitation laser itself causes the red-shift of the QD emission.

### Temporal and spectral signatures of re-excitation

In the experiment, we use an epitaxially grown In(Ga)As QD emitting in the telecom C-band, that is embedded in a circular Bragg grating cavity with wide-band enhancement and excited using a blue-detuned laser with adjustable pulse length. The QD transition is attributed to a charged exciton with a lifetime of *τ*_Q_ = 465 ± 1 ps, indicating a Purcell factor of 4.3 ± 0.8^51^. More details on the sample and laser preparation can be found in the Methods.

Starting with the temporal analysis of the re-excitation signal, we excite the QD with 80 ± 1 ps long Gaussian laser pulses, suppress the scattered laser and isolate the QD line from broadband background noise. The emission is detected in a Hanbury-Brown and Twiss (HBT) configuration and correlated with the laser clock (see inset Fig. 2b). Instead of performing a standard second-order autocorrelation *g*^(2)^(*τ*), we check for coincidences between the two optical channels and record the timestamp of the early and late click for each coincidence. Sorting this way, we separate events caused by 1^st^ photons from those caused by 2^nd^ photons, allowing us to resolve the dynamics of the two-photon emission individually. The resulting histograms, displayed in Fig. 2b, represent the temporal shape of the 1^st^ and 2^nd^ photon and thus provide the first direct experimental confirmation of the temporal ordering associated with the re-excitation process. Since the 1^st^ photon must be emitted when the laser is still present, its temporal profile strongly resembles that of the laser. The 2^nd^ photon, on the other hand, can be emitted during the full lifetime, which leads to the Gaussian-broadened exponential decay expected from spontaneous emission. Even though the probability distributions overlap in time, two photons originating from one laser pulse are always emitted into orthogonal time modes (see Supplementary Note 3) and only averaging over many repetitions induces the statistical overlap.

**Fig. 3:**
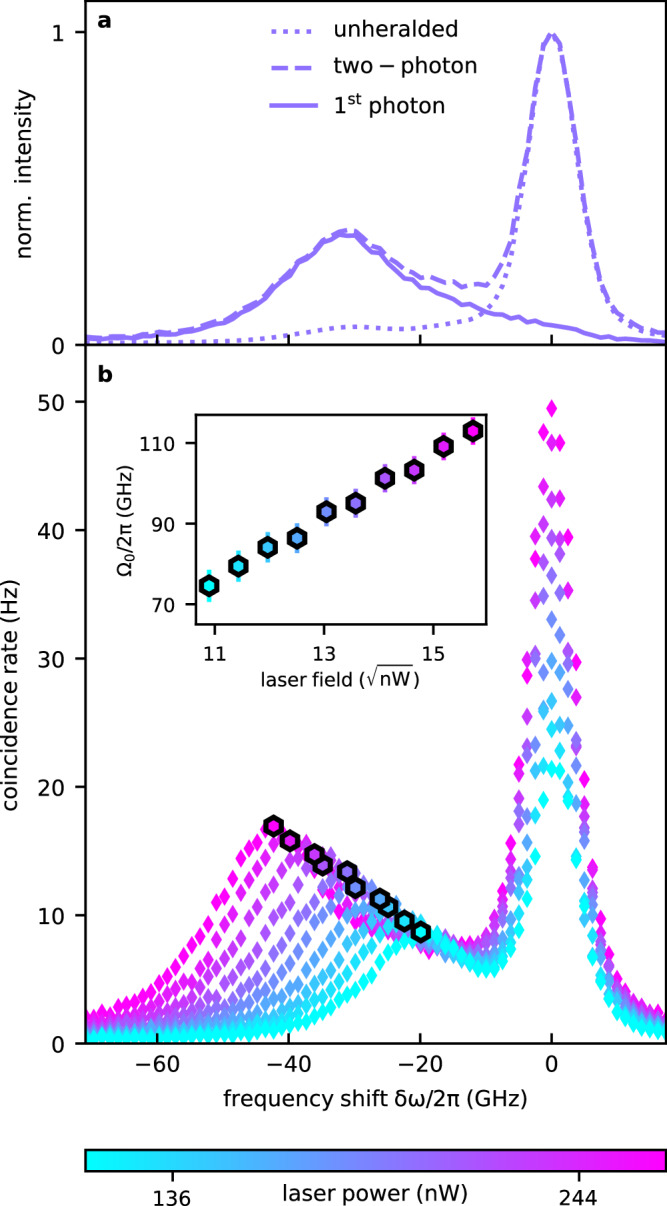
Re-excitation spectrum for varying pump power. A scanning frequency filter (*Δω*_f_/2*π* = 5.3 ± 0.3 GHz) is used to measure the QD spectrum based on different gating logics. Unheralded: no gating, all events detected on the spectrally filtered channel. Two-photon: all events on the spectrally filtered channel coinciding with a heralding photon within *Δτ*_c_ = 3 ns. 1^st^ photon: the heralding event is used only if it is registered >350 ps after the laser clock. The laser pulse length is *Δt*_L_ = 80 ± 1 ps and the detuning *δω*_L_/2*π* = 118 GHz. The displayed data is broadened by the transfer function of the frequency filter. **a** The laser power is set to 185 nW and individual data points have been omitted for visual clarity. **b** The laser power is varied (see color bar) and the two-photon spectrum (as above) is measured. The spectral position of the side peak is marked for every power and used to calculate the Rabi frequency according to Eq. (2), which is shown in the inset as function of the laser field. The inset error bars follow from the spectral resolution of the scanning setup.

Turning to the spectral analysis, we employ a tunable filter that enables us to frequency-resolve either the overall emission or, in a heralded manner, the two-photon signal (see inset Fig. 2b and Methods). Recording the count rate of the filtered path while scanning the filter frequency reconstructs the spectrum averaged over all photons, whereas using the second detector as herald post-selects on two-photon events, effectively measuring the two-photon spectrum (see Fig. 3a). The unheralded data features a prominent peak at *ω*_0_ representing undisturbed single-photon emission. However, because of the length of the laser pulses, the dressing effect is already noticeable without any heralding and shows itself in the broad shoulder on the low-frequency side, which we attribute to early photon emission. In the heralded configuration, where the spectra of the 1^st^ and 2^nd^ photon contribute equally, the shoulder then becomes a second but broader peak. To further support the assertion of the low-frequency peak to the 1^st^ photon, we refine the gating logic and ignore all heralding events occurring before *t* = 350 ps (compare Fig. 2b), that is, all photons emitted within the laser pulse duration. Counting coincidences in this way ensures that the photon in the filtered path must have been the 1^st^ photon. The resulting spectrum overlaps perfectly with the broad side peak observed in the two-photon spectrum (see Fig. 3a). The increased bandwidth of the 1^st^ photon is a consequence of its narrower temporal shape (recall Fig. 2b) and is also predicted for resonant driving^52^. The shift from the natural frequency *ω*_0_, on the other hand, is a signature of the transition between the dressed states $| \beta,N\rangle$ and $| \alpha,N-1\rangle$ and is unique to re-excitation under phonon-assisted pumping. Note that the cavity’s influence on these measurements is minimal, since its mode is much wider than the observed frequency shift (see Methods).

### Measuring the Rabi frequency for incoherent excitation

Following Eq. (1), we can express the maximum frequency shift as $\delta {\omega }_{\max }=\delta {\omega }_{{\mbox{L}}}-\sqrt{{\Omega }_{0}^{2}+\delta {\omega }_{{\mbox{L}}}^{2}}$. As expected from the dynamic Stark effect, the shift increases for stronger laser–QD interactions, that is, larger external fields or less detuning. Rearranging the equation as

2 $${\Omega }_{0}=\sqrt{\delta {\omega }_{\max }(\delta {\omega }_{\max }-2\delta {\omega }_{{\mbox{L}}})}$$

yields a remarkable result: we can infer the Rabi frequency—fundamental to coherent light–matter interaction in two-level systems—from an incoherent process. Instead of relying on Rabi rotations or coherent two-photon scattering (the origin of the Mollow-triplet^53^), here we exploit the signatures of the phonon-assisted re-excitation spectrum.

To experimentally verify these findings, we measure the two-photon spectrum for increasing laser power. Our results, presented in Fig. 3b, clearly demonstrate that the red-shift of the 1^st^ photon intensifies with power, while the spectral position of the 2^nd^ photon is unchanged. In the parameter regime studied here, and in agreement with the dressed-state model, the shift increases approximately linearly with the external laser field. Extracting *Ω*_0_ from the induced red-shift according to Eq. (2) and plotting it against the applied laser field shows an excellent linear trend (see inset Fig. 3b), confirming our understanding of the process. Note that coherent two-photon scattering, as recently shown also for pulsed excitation^54,55^, would produce a similar, but importantly, symmetric spectral response. And although both processes could coexist, we show in the Supplementary Note 5 that for the used laser parameters, efficient phonon coupling is ensured and the incoherent process dominates.

Lastly, we stress that the spectral overlap of the two photons reduces with increasing laser power. This is particularly helpful when frequency filtering the QD emission to reduce the multiphoton contribution, a method we introduce in the next section.

### Restoring high single-photon purities

Recalling that for applications of QDs as single-photon sources the presence of an additional 1^st^ photon diminishes the single-photon purity, we now leverage the strong spectro-temporal correlations found in Fig. 3 to our advantage. Using a frequency filter slightly wider than the spontaneous emission line at *ω*_0_, we expect to greatly suppress the 1^st^ photon. To ensure that we do not simply reduce the multiphoton noise caused by other spectrally distinct processes (such as neighboring QDs), we vary the laser pulse length across an order of magnitude while maintaining a constant laser power of 185 nW. In this way, we tune the re-excitation probability but leave other processes unaffected.

**Fig. 4:**
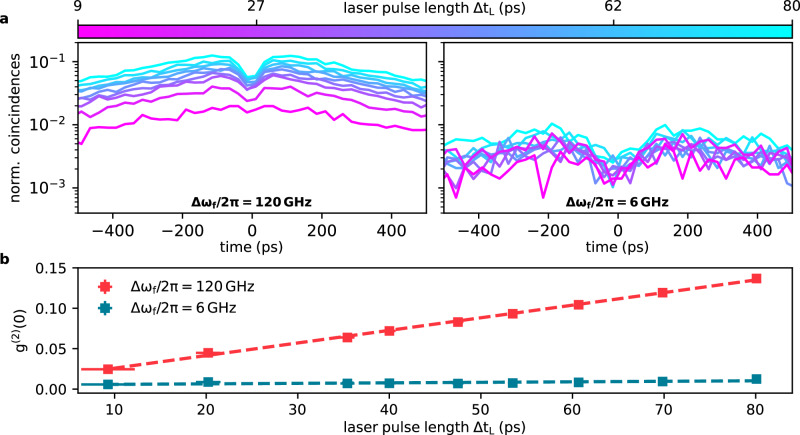
Reducing the multiphoton contribution by frequency filtering. For a constant laser power of 185 nW, the pulse length is varied and the second-order autocorrelation *g*^(2)^(*τ*) is measured for each step. **a** The zoomed-in central peak of the coincidence counts normalized to Poissonian level is shown as function of the delay *τ* for a 120 ± 1 GHz VBG filter (left panel) and a 6.0 ± 0.3 GHz etalon (right panel). **b** The *g*^(2)^(0) is evaluated by dividing the area of the correlated peak by that of the averaged uncorrelated peaks. The error bars are derived from Poissonian statistics and are too small to be visible. The uncertainty in laser pulse length arises from the deconvolution with the system response function of 26 ± 1 ps (see Methods). The linear fit *g*^(2)^(0, *Δt*_L_) = *A* ⋅ *Δt*_L_/*τ*_QD_ yields A_120 GHz_ = 0.72 ± 0.01 and A_6 GHz_ = 0.03 ± 0.01.

Figure 4a compares the zoomed-in, correlated peaks around *τ* = 0 of a standard *g*^(2)^(*τ*) for various pulse lengths. The QD signal shown in the left panel is only isolated from broadband background, but for the measurements displayed in the right panel a 6.0 ± 0.3 GHz etalon centered on *ω*_0_ is used. Unfiltered, the multiphoton probability clearly increases for longer pulses and the *g*^(2)^(*τ*) also features the characteristic re-excitation dip at *τ* = 0. The depth is limited by the system response function of the detection stage (see Methods). On the other hand, the filtered emission exhibits a drastically reduced multiphoton response and, importantly, is independent of the laser pulse length. Only for the longest pulse, a slight increase is notable. Since we maintain a constant laser power throughout the scan, the effective Rabi frequency decreases for longer pulses as ${\Omega }_{{{\mathrm{eff}}}}\propto 1/\sqrt{\Delta {t}_{{\mbox{L}}}}$, which results in a smaller red-shift of the 1^st^ photon and therefore in a larger spectral overlap with the spontaneous emission line. Interestingly, we also observe a (significantly wider) dip in the *g*^(2)^(*τ*) for the filtered signal, which could be caused by charge-carrier refilling^56^ or some residual transmission of the 1^st^ photon. This attribution is supported by time-resolved measurements (see Supplementary Note 6), which show that 1^st^ photons transmitted through the etalon are emitted at the very beginning of the laser pulse (when the dynamic frequency shift is still small), resulting in a larger delay between the 1^st^ and 2^nd^ photon.

The quantitative comparison of the unfiltered and filtered multiphoton probability, displayed in Fig. 4b, demonstrates the stark difference in *g*^(2)^(0) when the laser pulse length is tuned. The multiphoton component for the filtered emission is almost unaffected for all pulse durations studied here, reaching more than $1/6$ of the QD decay time. On the other hand, the *g*^(2)^(0) of the unfiltered signal increases linearly with *Δt*_L_, which is in good agreement with work on resonantly driven two-level systems^16^ and indicates that re-excitation dominates the multiphoton probability. Thus, this analysis shows that the proposed filtering method suppresses the re-excitation signal to the extent that its contribution to the *g*^(2)^(0) becomes negligible compared to other sources of noise, including detector dark counts and emission from neighboring QDs or the wetting layer. The filter and laser settings may be optimized depending on the application, which defines the ideal trade-off between single-and-multiphoton probabilities^31^. However, even modest effective Rabi frequencies as used here yield significant spectral separation, so it may be sufficient for most cases to set the laser parameters to maximize the excitation probability and use only the filter width to balance photon-number probabilities.

Finally, we note that the multiphoton response related to re-excitation scales with *Δt*_L_/*τ*_QD_, whereas the efficiency of the proposed filtering method depends on the frequency shift of the 1^st^ photon, which increases for shorter pulses (see above). Consequently, the filtering of multiphoton noise becomes more effective, benefiting sources with fast decay times in particular.

## Discussion

We have presented an experimental study that provides a nuanced understanding of the re-excitation dynamics and, at the same time, constitutes the first work on the phenomenon under phonon-assisted driving. In addition to verifying the strict temporal ordering of the emission predicted by theory^52^, we find strong spectro-temporal correlations for the 1^st^ emitted photon, which are induced by the dynamic Stark effect and lead to an asymmetric two-photon spectrum unique to phonon-assisted pumping. The implications of our results are both fundamental and practical. We have shown that the Rabi frequency of an incoherently driven QD can be extracted from the re-excitation spectrum, and on the application side, we have demonstrated how the spectral distinguishability of the two photons can be leveraged to efficiently remove multiphoton noise. We expect these results to have implications beyond QD single-photon sources; phonons are ubiquitous in solid-state platforms and phonon-assisted excitation has already been demonstrated for a range of quantum emitters^29,35,36^.

As advancements of high-Purcell cavities continue to reduce radiative lifetimes, managing the re-excitation signal with conventional methods becomes increasingly challenging, and the frequency filtering introduced here represents a compelling new approach. In applications, the laser pulses are significantly shorter than those used in this fundamental study, inducing a larger spectral shift, which allows for even higher transmission without compromising the single-photon purity. Moreover, our findings reveal an additional intrinsic advantage of phonon-assisted driving when used with narrow-band cavities. During the laser pulse, the dynamic frequency shift detunes the optical transition from the cavity mode, effectively cancelling the Purcell enhancement. This mechanism delays photon emission until the laser pulse subsides and greatly suppresses re-excitation.

We have shown that a deeper understanding of phonon-assisted excitation offers unique advantages for applications; however, other key aspects of the exciton–phonon interactions are yet to be explored. In particular, the impact of the phonon spectral density, which can be tailored^57^ using phononic cavities, presents a promising research direction that could create new opportunities in quantum communication and photonic quantum computing.

Finally, analyzing the re-excitation sensitivity in other excitation schemes using the framework of temporally evolving dressed states is a promising direction; coherent methods, such as adiabatic rapid passage^58^–where the laser chirp introduces a temporal asymmetry–or the SUPER scheme^59^–which can be understood as doubly dressing the two-level system^60^–are likely to show more favorable scaling than resonance fluorescence.

During the submission process, we became aware of related work focusing on re-excitation under resonant excitation and exploring the efficiency of spectral filtering purely based on the different line shapes of the 1^st^ and 2^nd^ photon^61^.

## Methods

### Quantum dot sample

The single-photon source is based on an epitaxially grown In(Ga)As quantum dot coupled to a circular Bragg grating cavity with a FWHM of ≈ 1250 GHz. Starting from a GaAs substrate, a non-linear metamorphic buffer layer^62^ is deposited. Reducing the lattice mismatch to the subsequently grown InAs QD layer enables emission in the telecom C-band. In the next step, circular Bragg grating cavities are etched into the sample in a non-deterministic process. More details on the fabrication and the exact geometry of the final structure can be found in ref. ^63^ and the corresponding supplementary material, following the same design principles. The QD transition is attributed to a charged exciton that features highly polarized emission (>95%)^51^ at *λ*  =  1552.25 ± 0.03 nm. Under optimized LA phonon excitation conditions for a ≈ 20 ps pulse (≈ 250 nW laser power at *δω*_L_/2*π* ≈ 120 GHz), we achieve a polarized and fiber-coupled single-photon generation efficiency of (5.7 ± 0.2)% at *g*^(2)^(0) = 0.013 ± 0.001. The spontaneous emission line is subject to spectral diffusion and not Fourier-transform limited. The inhomogeneous broadening results in a predominantly Gaussian line shape. The measurement data used to infer the lifetime and cavity mode width can be found in the Supplementary Note 2.

### Temporal measurements and autocorrelation

The system response function (SRF) characterizes how the measurement apparatus blurs the detection time of an infinitely short pulse. In our setup, the jitter introduced by the superconducting nanowire single-photon detectors (SNSPDs, Single Quantum EOS) dominates compared to the time-tagging device (Swabian Time Tagger X). From a correlation between the electrical laser clock and an SNSPD channel exposed to an attenuated 1.8 ps long laser pulse, we measure the SRF as full width at half maximum (FWHM), yielding 26 ± 1 ps for the first and 29 ± 1 ps for the second channel. The timing jitter for an autocorrelation between both optical channels is 39 ± 2 ps.

From the second-order autocorrelation functions *g*^(2)^(*τ*) shown in Fig. 4a we evaluate the multiphoton probability *g*^(2)^(0) presented in Fig. 4b by dividing the area of the peak around *τ* = 0 by the averaged area of the neighboring peaks at *τ* = ± 13.17 ns. Due to electronic or optical signal reflections observed at ≈ ± 5 ns that should not be included in the multiphoton estimation, we use an integration window of 6.58 ns, which corresponds to half the repetition interval of the experiment. We note that this integration time still captures >99.9% of the emitted photons.

### Laser pulse generation

Starting from 9 ± 3 ps long approximately Gaussian laser pulses generated at a repetition rate of 75.95 MHz by a fiber-based laser (Pritel UOC), we use a tuneable width frequency filter (EXFO XTM-50) to reduce the spectral width, thereby stretching the pulse. The smallest possible bandwidth of the filter is measured using a tuneable cw-laser (Santec TSL550) and is 5.7 ± 0.3 GHz. For each bandwidth setting, the resulting pulse length is measured by sending a strongly attenuated pulse to the SNSPD and deconvolving the detected signal using the SRF.

### Spectral measurements

The QD emission is passed through a volume Bragg grating (VBG) that reflects the scattered laser light with an FWHM of 120 ± 1 GHz and OD6. A second identical VBG, centered at ≈ −40 GHz with respect to the spontaneous emission frequency *ω*_0_, selects the QD transition under study and isolates it from any remaining broadband background.

Requiring low dark count noise and a gating logic, the analysis setup is based on a scanning frequency filter and photon detection using SNSPDs and a time tagger. The scanning filter consists of a cascaded pair of etalons with a free spectral range (FSR) of 125 GHz (292 GHz) and FWHM of 5.5 ± 0.3 GHz (16.4 ± 0.3 GHz). Using piezomotor-controlled angle tuning, the resonance frequencies are overlapped and then jointly scanned. The spectral resolution of the setup of 5.3 ± 0.3 GHz is dominated by the narrow-band etalon. The two-photon spectrum is measured by correlating (coincidence window *Δτ*_c_ = 3 ns) the filtered and unfiltered channel while tuning the central frequency stepwise. In this way, plotting the coincidence count rate as a function of the filter position reconstructs the two-photon spectrum. The 1^st^ photon spectrum is acquired in the same way, but the heralding channel is additionally gated in postselection, such that only clicks registered within 350 ps < *t* < 3000 ps after the laser clock signal are accepted and used for the subsequent correlation with the filtered optical channel.

## Supplementary information

Supplementary Information

Transparent Peer Review file

## Source data

Source Data

## Data Availability

The raw time-tag data that support the findings of this study exceed storage and file number limitations of general repositories, but are available from the corresponding author upon request. Source data are provided with this paper.
