# Supplementary material for: Asymmetric two-photon response of an incoherently driven quantum emitter
Source: Nat Commun. 2026 May 9;17:6400. doi: 10.1038/s41467-026-72706-z (PMC13376212; doi:10.1038/s41467-026-72706-z)
Supplement: Supplementary file 1 — Supplementary Information [file 41467_2026_72706_MOESM1_ESM.pdf]

Asymmetric two-photon response of an incoherently driven quantum emitter

Lennart Jehle,[1,2,*] Lena M. Hansen,[1,2] Patrik I. Sund,[1,2] Thomas W. Sandø,[1,2] Raphael Joos,[3] Michael Jetter,[3] Simone L. Portalupi,[3] Mathieu Bozzio,[1] Peter Michler,[3] and Philip Walther[1,2,4]

[1] *University of Vienna, Faculty of Physics, Vienna Center for Quantum Science and Technology (VCQ), 1090 Vienna, Austria*
[2] *Christian Doppler Laboratory for Photonic Quantum Computer, University of Vienna, Faculty of Physics, 1090 Vienna, Austria*
[3] *Institut für Halbleiteroptik und Funktionelle Grenzflächen, Center for Integrated Quantum Science and Technology (IQST) and SCoPE, University of Stuttgart, Allmandring 3, 70569 Stuttgart, Germany*
[4] *Institute for Quantum Optics and Quantum Information (IQOQI) Vienna, Austrian Academy of Sciences, Vienna, Austria*

Supplementary Note 1. PHONON-ASSISTED EXCITATION IN THE DRESSED-STATE MODEL

By modeling a quantum dot (QD) as a two-level system (TLS) with a ground state $|G\rangle$ and an excited state $|X\rangle$ that is $\hbar\omega_0$ higher in energy, the interaction with a monochromatic laser field of frequency ω_L can be described by the Hamiltonian

$$H = \hbar\omega_0 |X\rangle\langle X| + \hbar\omega_L(a^\dagger a + 1/2) + H_{QD-L}\,, \tag{1}$$

where $a^\dagger$ and a are the photonic creation and annihilation operators and H_{QD-L} is the QD-laser coupling. We denote $\delta_L = \omega_L - \omega_0$ as the laser-QD detuning, and for near-resonance interactions studied here $|\delta_L| \ll \omega_0$.

For $H_{QD-L} \to 0$, the eigenstates of Eq. (1) may be written as product states of the TLS and the laser field. The spectrum of eigenstates can be divided into N subspaces, where N denotes the number of photons present in the laser field. Each subspace, or manifold, consists of a ground state $|G, N+1\rangle$ and an excited state with one laser photon less $|X, N\rangle$ that are $\hbar\delta_L$ apart from each other

$$\mathcal{E}(N) = \{|G, N+1\rangle\,, |X, N\rangle\}\,. \tag{2}$$

Neighboring subspaces $\mathcal{E}(N)$ and $\mathcal{E}(N-1)$ have an energy difference of $\hbar\omega_L$.

To include the interaction potential, the dipole and rotating wave approximations are applied, such that the Hamiltonian may be written as

$$H_{QD-L} = \frac{\hbar}{2}\left(\Omega^*(t)|G\rangle\langle X| + \Omega(t)|X\rangle\langle G|\right)\,, \tag{3}$$

where $\Omega(t)$ is the Rabi frequency, which is proportional to the driving laser field.

For each manifold, H_{QD-L} gives rise to a new set of eigenstates, the so-called laser-dressed states, that can be seen as hybrid atom/field states. The high-energy (low-energy) state is conventionally denoted by α (β)

$$|\alpha, N\rangle = \cos(\vartheta)|G, N+1\rangle - \sin(\vartheta)|X, N\rangle \tag{4}$$

$$|\beta, N\rangle = \sin(\vartheta)|G, N+1\rangle + \cos(\vartheta)|X, N\rangle \tag{5}$$

with the admixing angle

$$\vartheta = \frac{1}{2}\arctan\left(\frac{\Omega(t)}{\delta_L}\right)\,. \tag{6}$$

Eqs. (4)-(5) assume a blue-detuned driving laser ($\delta_L > 0$), but one finds a similar set for $\delta_L < 0$. Due to the cryogenic temperatures in the experiment and their effect on the phononic interactions introduced below, we restrict our description of the dressed states to positive detunings from hereon.

* Address all correspondence to lennart.jehle@univie.ac.at

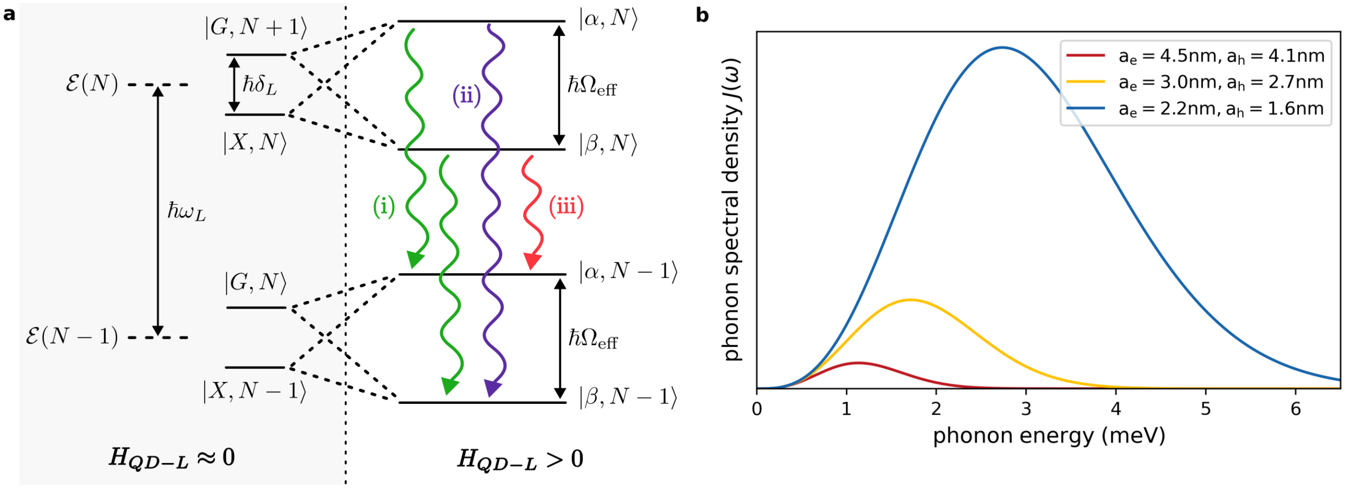

Supplementary Figure 1. **Energy scheme of dressed TLS and phonon spectral density.** **(a)** In the unpertubed basis, only transitions $|X,N\rangle \to |G,N\rangle$ are dipole-allowed but since both dressed states of the upper (lower) manifold have a $|X,N\rangle$ ($|G,N\rangle$) admixture, all four inter-manifold transitions of the dressed states are possible. There are two degenerate transitions (i) at $\omega_e = \omega_L$ and one at either the high (ii) and low (iii) frequency side $\omega_e = \omega_L \pm \Omega_{\text{eff}}$. For simplicity, the laser drive is time independent. **(b)** Assuming spherically symmetric wave functions for the electron and hole, the exciton-phonon interaction is shown for three sets of radii as indicated by the legend. The spectral density is follows from Eq. (9) using the following parameters: speed of sound $c_s = 4200\,\text{m/s}$, density $\rho = 5680\,\text{kg/m}^3$, electron potential $D_e = 3\,\text{eV}$, hole potential $D_h = -6\,\text{eV}$.

The dressed-state level splitting $\hbar\Omega_{\text{eff}}(t)$ is given by the effective Rabi frequency

$$\Omega_{\text{eff}}(t) = \sqrt{\Omega(t)^2 + \delta_L^2} \tag{7}$$

and for a vanishing laser drive $\Omega \to 0$, $|\alpha, N\rangle$ ($|\beta, N\rangle$) transform back to the ground (excited) state of the TLS.

All four transitions between dressed states of neighboring manifolds are allowed by optical selection rules (see Fig. S1a) and for a cw laser drive, these give rise to the well-known Mollow-Triplet[1]. Importantly, without any phonon or cavity coupling, the energy transitions at $\omega_L \pm \Omega_{\text{eff}}$ have the same spectral weight due to the conservation of energy. However, phononic interactions can activate transitions between dressed states within the *same* manifold and lead to significant changes in the optical spectrum. We include the phonon coupling using

$$H_{QD-P} = \sum_{\boldsymbol{q}} \hbar\omega_{\boldsymbol{q}} b_{\boldsymbol{q}}^\dagger b_{\boldsymbol{q}} + \sum_{\boldsymbol{q}\nu} \hbar n_\nu \left(\gamma_{\boldsymbol{q}} b_{\boldsymbol{q}} + \gamma_{\boldsymbol{q}}^* b_{\boldsymbol{q}}^\dagger\right) |\nu\rangle \langle\nu| \;, \tag{8}$$

where $b_{\boldsymbol{q}}^\dagger$ ($b_{\boldsymbol{q}}$) creates (annihilates) a phonon with frequency $\omega_{\boldsymbol{q}}$ and wave vector $\boldsymbol{q}$, n_ν counts the excitons in state $|\nu\rangle$ and γ_q denotes the electron-phonon coupling constants. In general, various interactions with optical or acoustic, longitudinal or transversal, surface or bulk phonons may be captured[2] by γ_q, but for the purpose of this work, the description can be limited to longitudinal acoustic (LA) phonons coupled to the QD via deformation potentials of electrons D_e and holes D_h. It was shown that the exact shape of the electron and hole wave function only slightly influences the phonon interaction spectrum[3], and in later experiments, good agreement was obtained using spherically symmetric wave functions[4] with radius $a_{e/h}$. The spectral density of the phonon coupling then reads as[5]

$$J(\omega) = \sum_{\boldsymbol{q}} \gamma_{\boldsymbol{q}} \delta(\omega - \omega_{\boldsymbol{q}}) = \frac{\omega^3}{4\pi^2 \rho \hbar c_s^5} \left(D_e e^{-\omega^2 a_e^2/(4c_s^2)} - D_h e^{-\omega^2 a_h^2/(4c_s^2)} \right) \tag{9}$$

with the mass density ρ and c_s as the speed of sound. Exemplary spectral densities using typical QD parameters are shown in Fig. S1b.

Including the phonon interaction, the LA phonon-assisted excitation mechanism can be understood as follows. Dynamically dressing the TLS with an external field transforms the ground state into the upper dressed state, and initially $|\alpha, N\rangle$ is predominantly populated because of the large $|G, N+1\rangle$ component. At the same time, phonon

interactions activate population exchange between the states of the *same* manifold, $|\alpha, N\rangle$ and $|\beta, N\rangle$. Due to the low occupation of phonon modes at cryogenic temperatures, the creation of a phonon is much more likely than absorbing one, strongly favoring the transition $|\alpha, N\rangle \rightarrow |\beta, N\rangle$ compared to the reverse transition. The imbalance drives the population into the low-energy state (also called thermalization). At elevated temperatures, the asymmetry in the transition rates weakens, and the upper dressed state retains some residual population. Simulations suggest that for 50 K to 77 K the occupation probability of both dressed states tends to 0.5[6,7], thus limiting the excitation efficiency of phonon-assisted pumping. At cryogenic temperatures on the other hand, excitation probabilities of >90 % are feasible[5]. Using Fermi's Golden rule, the phonon scattering rate[8]

$$\Gamma_{\text{phonon}}(t) = \frac{\pi}{4} \frac{\Omega(t)}{\Omega_{\text{eff}}(t)} J\left[\Omega_{\text{eff}}(t)\right] , \tag{10}$$

can be used to estimate the transition probability for a given set of laser and QD parameters, and understand its dynamics. Eq. (10) indicates that at any point in time, the relaxation rate to the lower dressed state is proportional to the phonon spectral density $J(\omega)$ evaluated at the instantaneous effective Rabi frequency. Consequently, there is an optimal driving strength for each detuning δ_L that maximizes the exciton-phonon interaction. At the end of the ideal laser pulse, almost only the lower dressed state is populated, which, as the laser field decreases, is adiabatically transformed back to the excited state of the TLS[5,9].

However, due to the finite pulse lengths, there is a probability that the population of lower dressed state transitions to the upper dressed state of the neighboring manifold $|\beta, N\rangle \rightarrow |\alpha, N-1\rangle$ while the laser field is still present (the transition probability for $|\beta, N\rangle \rightarrow |\beta, N-1\rangle$ is much smaller for the laser power and detunings used here). The frequency of the emitted photon corresponds to the energy separation *at this time*, that is

$$\omega_e(t) = \omega_L - \Omega_{\text{eff}}(t) . \tag{11}$$

Importantly, if there is still significant phonon coupling after the photon emission, the system can relax once again to the lower dressed state $|\alpha, N-1\rangle \rightarrow |\beta, N-1\rangle$ and thus subsequently cause the emission of a second photon as schematically depicted in Fig. 2a of the Main Text.

Supplementary Note 2. EXPERIMENTAL SETUP

The experimental setup is shown in Fig. S2. We use an Er-doped fiber mode-locked pulsed laser (*PriTel UOC*) to excite the quantum dot (QD) at a repetition rate of $\nu_{\text{rep}} = 75.95$ MHz. A filter with 1 nm bandwidth placed within the laser cavity stretches the generated pulses to a temporal pulse width of (9 ± 3) ps (spectral width FWHM ≈ 450 pm, the pulses are not fully Fourier-limited). The laser provides wavelength tunability between 1530 nm and 1555 nm at an average output power of 200 mW. All remaining broadband amplifier noise is removed by a free-space 4-f pulse shaper based on a reflective grating (*Spectrogon*, 1200 lines/mm, blaze at 1550 nm) with an efficiency of ≈ 90 %, C-coated cylindrical lens with a focal length of 500 mm and a variable filtering slit. Finally, a fiber-coupled tunable bandwidth filter (*EXFO, XTM-50*) with a top-hat profile is used to stretch the pulses. The minimal bandwidth is (5.7 ± 0.3) GHz and results in a maximum pulse length of (80 ± 1) ps that is independently verified using a superconducting nanowire single-photon detector (SNSPD, *Single Quantum EOS*) and a time-tagging device (*Swabian Time Taggers TimeTagger X*).

The pulse-shaped excitation beam is collimated into free space by an 8 mm lens collimator (*Schäfter+Kirchhoff 60FC-SF-0-M08-08*). A Glan-Taylor polarizer (*Leysop GTB10-M*, polarization extinction ratio > 60 dB) sets the excitation beam polarization to linear and the angle of the polarization is adjustable by a subsequent half-wave plate (*Bernard Halle*). Just before the cryostat chamber, a 90:10 beam splitter cube (BS, *Thorlabs BS078*) is placed to separate the incoming excitation beam from the single photons emitted by the QD. Approximately 90 % of the excitation beam (depending on its polarization state) is reflected by the BS to a power meter (used to control the QD excitation power) and only ≈ 10 % of the laser power is guided to the cryostat chamber (*attocube attoDry800*) in which the QD sample is placed. In Fig. S3a, the cavity mode of the circular Bragg grating is shown and we infer a FWHM of ≈ 1250 GHz. Fitting the time-correlated photon-counting measurement presented in Fig. S3b yields a lifetime of $\tau_{QD} = (465 \pm 1)$ ps. For more details on the QD sample see Methods of the Main Text.

The QD emission and the reflected laser light are collected by a lens ($f = 3.1$ mm) with an NA of 0.68. Leaving through the reflection port of the 90:10 BS, QD emission and laser light are separated using a volume Bragg grating (VBG) notch filter (*OptiGrate*) that blocks the laser wavelength with a spectral bandwidth (FWHM) of (120 ± 1) GHz and an individual suppression of OD6. Finally, the QD emission is filtered out from the remaining broadband

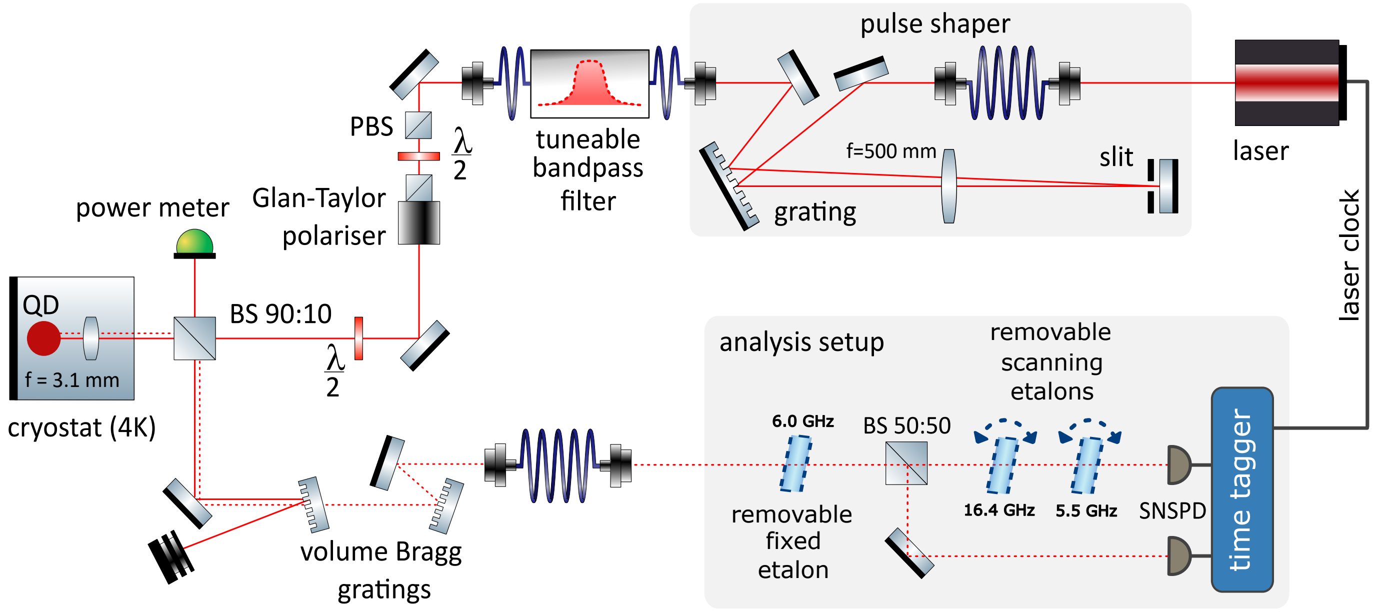

Supplementary Figure 2. **Scheme of the experimental setup.** A schematic representation of the main building blocks of the experimental setup, such as the pulsed excitation laser, pulse shaper, tuneable bandwidth filter, excitation of the quantum dot (QD) sample, filtering of the QD transition line and the analysis stage. Solid (dashed) red lines indicate the path of the laser beam (QD emission). PBS: polarizing beam splitter, BS: beam splitter, SNSPD: superconducting nanowire single-photon detector. The figure has been modified with permission under CC-BY-4.0 license from previous work by the authors[10].

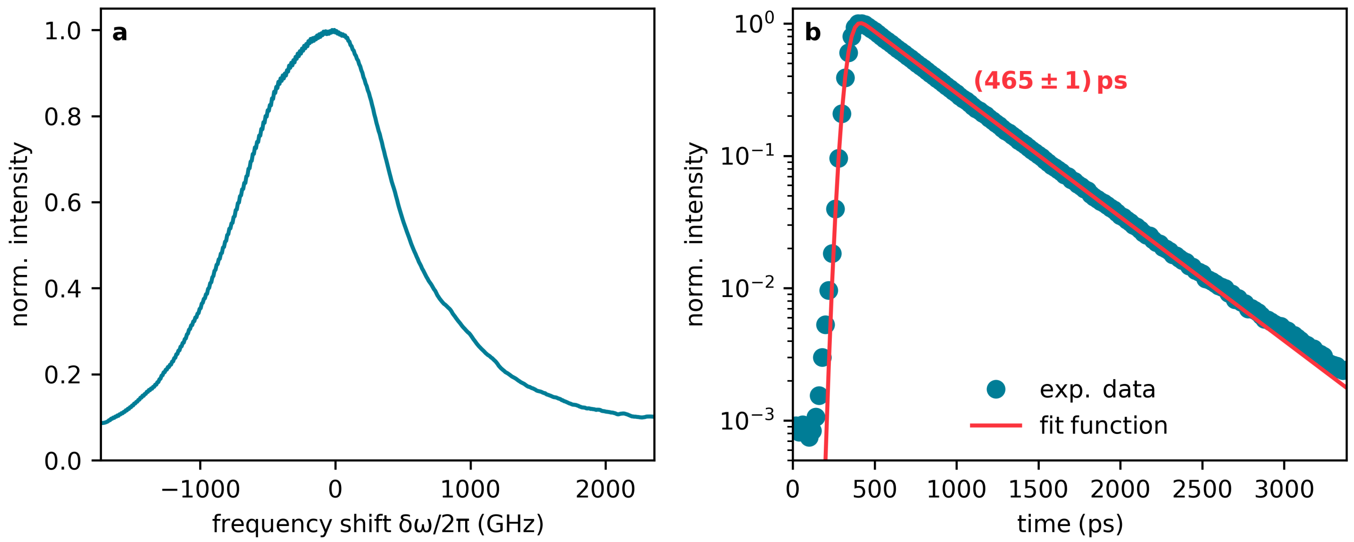

Supplementary Figure 3. **Characteristics of the QD sample.** **(a)** The cavity mode is measured using a strong above band laser and collecting the emission of the sample on a spectrometer. The frequency is given as detuning from the spontaneous emission line, that is $\delta\omega = \omega - \omega_0$. The spectrometer resolution is $\approx$10 GHz per pixel. **(b)** The measurements are performed with pulsed LA excitation at 75.95 MHz, a blue-shifted detuning of 175 GHz, a laser spectral bandwidth of (26 ± 1) GHz and excitation power of (250 ± 15) nW. The mono-exponential fit function (solid black line) yields a decay time of (465 ± 1) ps.

spectrum by reflecting of another VBG (*OptiGrate*) centered at $\approx \omega_0/2\pi - 40\,\text{GHz}$ and coupled by a $f = 8\,\text{mm}$ lens collimator into a single-mode fiber (SMF).

The analysis setup is arranged in a Hanbury-Brown and Twiss (HBT) configuration and can be equipped with additional frequency filters, depending on the desired measurement type. All spectrally resolved measurements employ a set of two etalons (FSR: $(125 \pm 1)\,\text{GHz}$ and $(292 \pm 1)\,\text{GHz}$; FWHM: $(5.5 \pm 0.3)\,\text{GHz}$ and $(16.4 \pm 0.3)\,\text{GHz}$), each mounted on a closed-loop piezomotor-controlled rotation stage *(attocube ECR5050hs/Al/RT)* to scan their central frequency jointly, which are placed in one of the two HBT paths. The conversion from an angle to a frequency dependence is performed based on a calibration table previously recorded using a cw laser with a linewidth of 20 kHz. The cascaded arrangement of two etalons with non-integer-divisible FSRs greatly suppresses spectral components outside the overlapped frequency peak. The overall frequency repeatability of the analysis setup is in the few MHz range as estimated from the angle repeatability of the piezo stage and the angle tunability of the etalons. For the spectrally filtered autocorrelation presented in Fig. 4 of the Main Text, the 50:50 BS is preceded by a $(6.0 \pm 0.3)\,\text{GHz}$ Lorentzian etalon fixed at ω_0, but the scanning etalons are removed.

Supplementary Note 3. TEMPORAL MODES OF TWO-PHOTON EMISSION

When using the wording *two-photon emission* in the Main Text, we refer to the fact that two photons are emitted during or shortly after the interaction of QD with the laser pulse. However, these photons are never emitted simultaneously (that is, in the same time mode), and the overlap in arrival times of the 1st and 2nd photon (as observed in Fig. 2b of the Main Text) is the result of statistical averaging over many repetitions. These arrival-time distributions indicate that for some point in time, a detection could have been caused by either a 1st or a 2nd photon, but they do not imply that two photons have been emitted simultaneously.

To demonstrate this, we display the time-resolved autocorrelation function $G(t_1, t_2)$ in Fig. S4, where t_1 (t_2) is the arrival time measured in the first (second) optical channel with respect to a timing reference, typically the electronic clock of the pulsed laser. Each coincidence is assigned to a bin in the histogram according to the arrival time of both photons. The diagonal in the histogram corresponds to events that occur at $t_1 = t_2$, such that it reflects the probability that two photons arrive at the same time (more accurately, within the binning width). Importantly, the diagonal shows significantly lower counts than the surrounding bins, indicating that two photons are unlikely to be

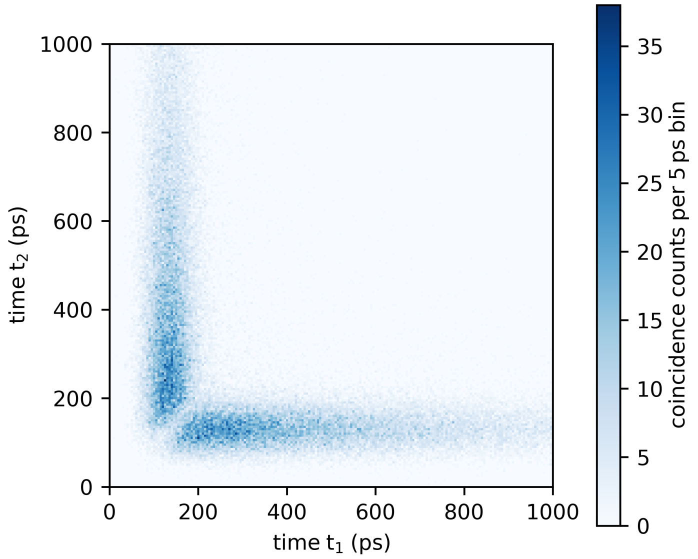

Supplementary Figure 4. **2D histogram.** The two-photon coincidences are displayed in the 2D histogram with a bin width of 5 ps per time axis. The long laser pulses are $(80 \pm 1)\,\text{ps}$ long, have a cw-power of 250 nW and a detuning of $\delta\omega_L/2\pi = 125\,\text{GHz}$, The emission from the QD was isolated from broadband background using a $(120 \pm 1)\,\text{GHz}$ VBG.

emitted at the same time. Residual coincidences on the diagonal are caused by the finite system response function of (39 ± 2) ps of the detection setup, background emission and detector dark counts.

Supplementary Note 4. CORRELATION LOGIC

The correlation method for extracting individual photon shapes from the raw time tags (see Main Text, Fig. 2b) is not widely used and is thus explained in more detail here. From the three physical signal streams (laser reference clock and two SNSPDs), two virtual channels are created in an intermediate step: an early (1st) and a late (2nd) coincidence channel. Whenever two optical signals are registered within a window of 2 ns, they are accepted as a coincidence, and the signal clicks comprising it are allocated to the respective coincidence channel as illustrated in Fig. 5. Then, histograms are calculated between the laser reference and the two coincidence channels, representing the temporal shapes of the early (1st) and late (2nd) components of each two-photon event. Since each physical channel is only detected with finite timing precision (see Methods), a fraction of those coincidence events is allocated to the wrong channel. In the following, we estimate this systematic error.

A misallocation requires that photon 1 at detector A is registered before photon 2 on detector B, even though in true arrival times (that is, before the jitter of the measurement apparatus skews the timing), photon 1 arrived after photon 2. The system response function $\mathrm{SRF}(\tau')$ for the correlation of the two physical channels used here is obtained using a short ($\approx$1.8 ps) attenuated laser pulse, and its FWHM is found to be (39 ± 2) ps. The SRF can be interpreted as the probability that two photons are registered with a timing difference of τ' even though they arrived simultaneously at their respective detectors. Consequently, the probability that the temporal ordering of a coincidence event in the main experiment was reversed due to detection jitter can be bounded using the SRF. For a coincidence with a time difference of τ the probability that it was falsely allocated is given by the cumulative probability $p_{\mathrm{err}}(\tau) = \int_{\tau}^{\infty} d\tau'\ \mathrm{SRF}(\tau')$. Overlapping p_{err} with the second-order correlation $G^{(2)}(\tau)$ recorded in the experiment allows us to upper-bound the systematic error p_{sys} as illustrated in Fig. S6 (since both $\mathrm{SRF}(\tau')$ and $G^{(2)}(\tau)$ are symmetric with respect to τ we limit us to $\tau > 0$). The systematic error follows as

$$p_{\mathrm{sys}} = \frac{\int_0^{2\,\mathrm{ns}} d\tau\ p_{\mathrm{err}}(\tau) G^{(2)}(\tau)}{\int_0^{2\,\mathrm{ns}} d\tau\ G^{(2)}(\tau)} < 0.8\,\% \,. \tag{12}$$

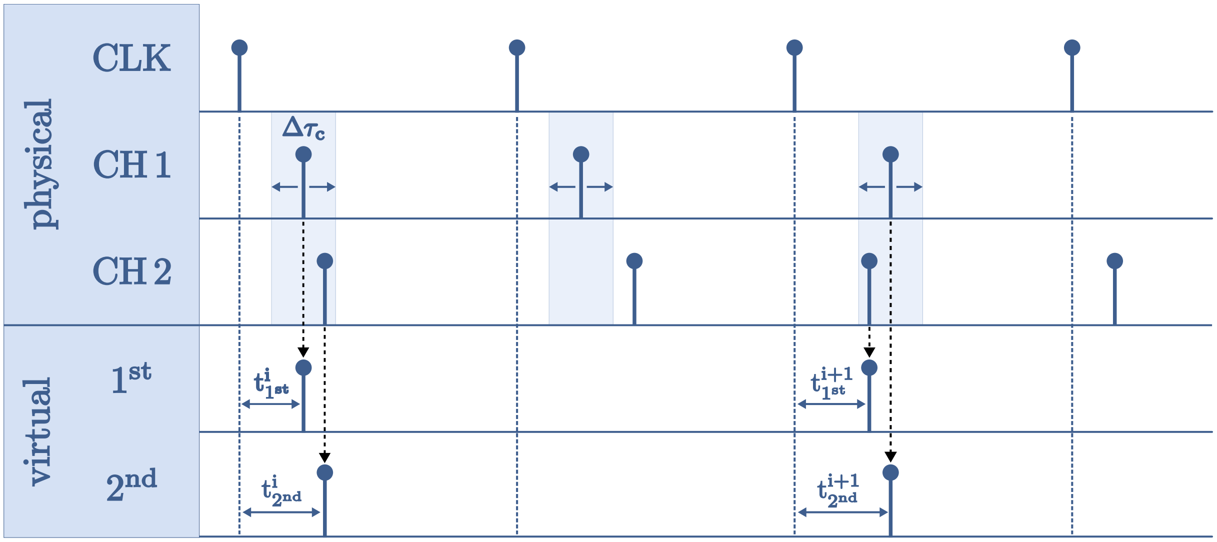

Supplementary Figure 5. **Schematic of the correlation logic.** The time tagger records three physical channels: the reference clock (CLK) provided by the pulse laser, and the electronic output of two SNSPDs (CH 1 and CH 2. Whenever two events on the optical channels are registered within a coincidence window $\Delta\tau_c = 2$ ns, they are accepted as coincidence, and the individual events are onto the virtual channels where the early click of each pair is assign to one channel (1st) and the late click to the other (2nd). The histograms between the reference clock and the virtual channels then represent the temporal shapes of the individual photons as displayed in Fig. 2b of the Main Text.

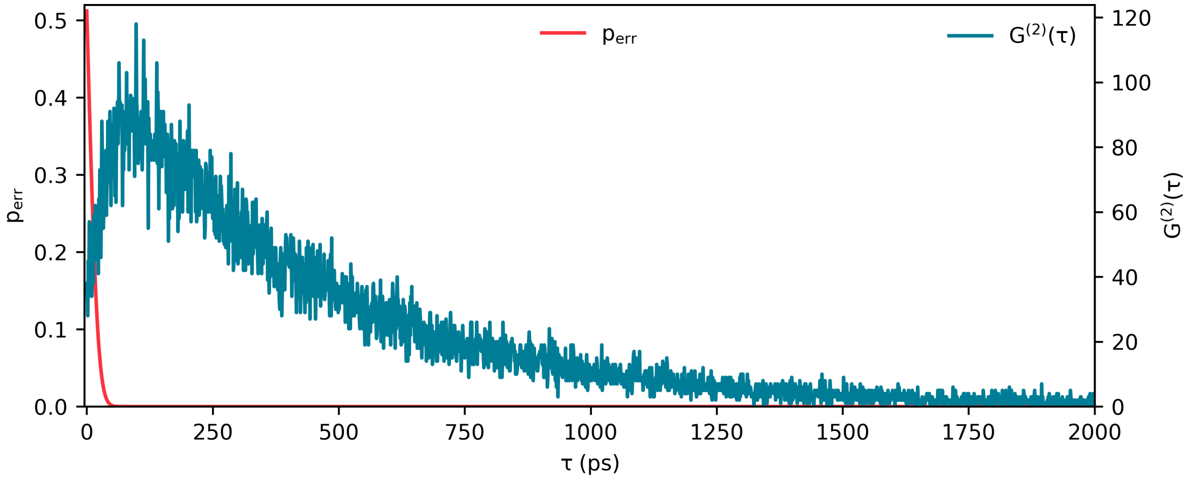

Supplementary Figure 6. **Analysis of systematic assignment errors.** From the system response function (SRF) of the two correlated optical channels, the probability of falsely recording the order of events that are τ apart is at most $p_{\mathrm{err}}(\tau) = \int_{\tau}^{\infty} d\tau' \; \mathrm{SRF}(\tau')$. Considering how many coincidence events were detected for each time difference, which is represented by $G^{(2)}(\tau)$, allows us to bound the overall probability p_{sys} that any given coincidence event was falsely allocated. The majority of coincidences were detected when $p_{\mathrm{err}}(\tau)$ is essentially zero, and thus $p_{\mathrm{sys}} < 0.8\,\%$. The $G^{(2)}(\tau)$ is calculated from the same dataset as shown in Fig. 2b of the Main Text.

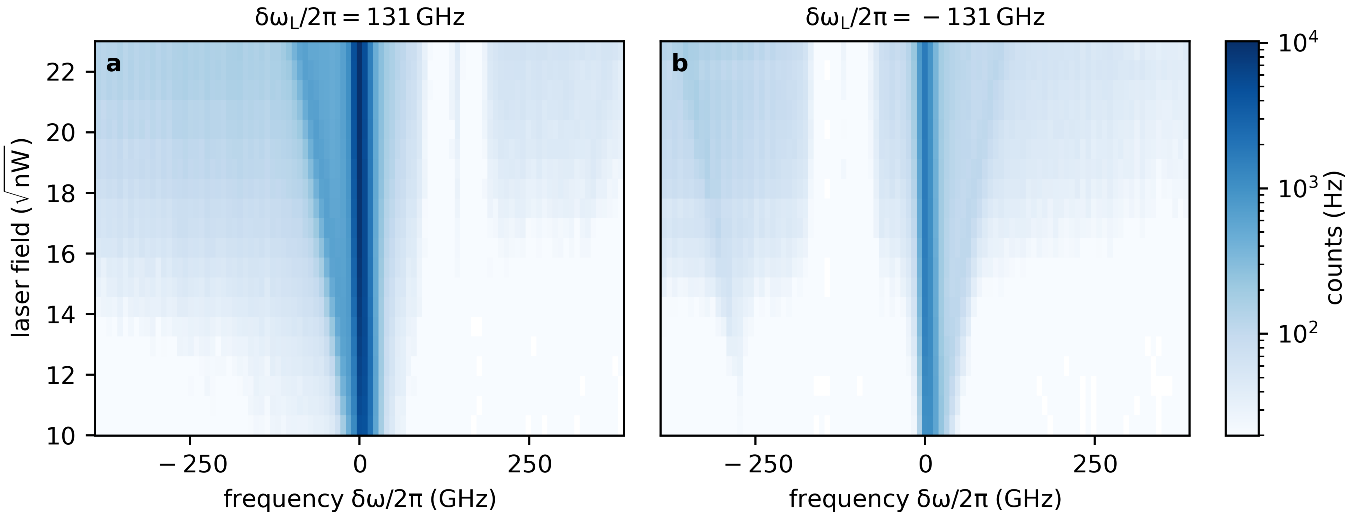

Supplementary Figure 7. **QD spectrum for blue- and red-detuned laser at varying powers.** For (80 ± 1) ps long laser pulses, the QD emission is measured with a spectrometer after the scattered laser is filtered using a (120 ± 1) GHz VBG. The spectrometer resolution is $\approx$10 GHz per pixel. **(a)** For a 131 GHz blue-detuned laser, only the low-frequency side peak is visible, whereas the emission for a red-detuned excitation laser (**b**) features two side peaks, that are symmetric with respect to the laser frequency.

Supplementary Note 5. EXTENDED DATA SET

As discussed in the Main Text, other signatures of dynamically dressed states were recently observed[11,12]. These experimental findings represent the extension of the well-known Mollow-triplet[1] into the regime of pulsed driving and bear some similarity to the spectra presented here. However, the underlying processes are fundamentally different.

As explained in Supplementary Note 1, there are four optical transitions between any two manifolds of laser-dressed

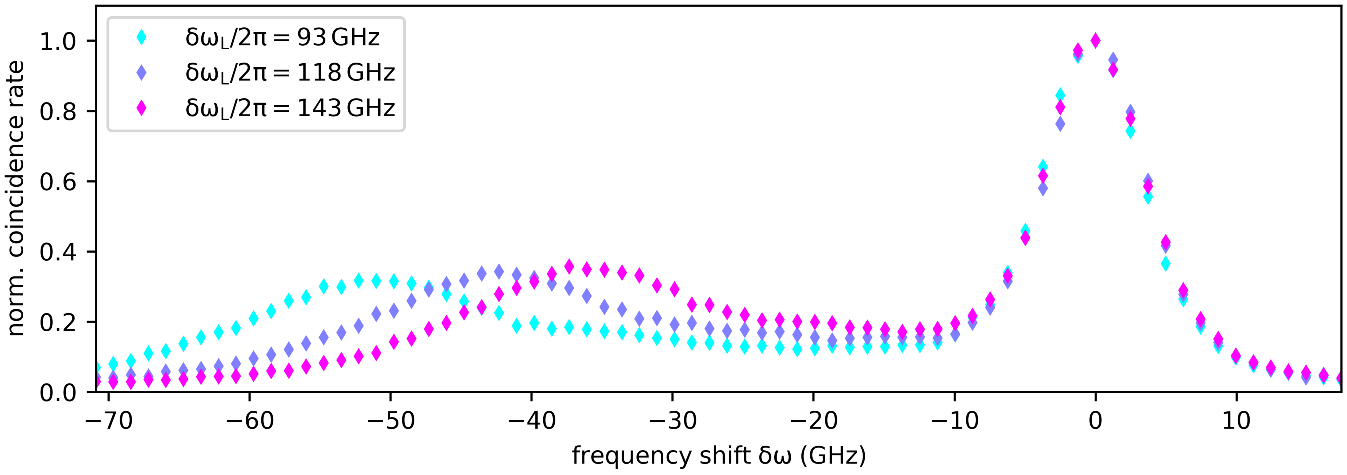

Supplementary Figure 8. **Two-photon spectrum for varying laser–QD detunings.** For (80 ± 1) ps long laser pulses with 250 nW, the two-photon spectrum is measured in the heralded configuration explained in the Main Text. The three measurements represent different laser–QD detunings $\delta\omega_L$. For clarity, all error bars were omitted.

states, all of which are allowed by optical selection rules[13]. Two of them are degenerate and match the frequency of the driving laser, the other two appear at $\omega_L \pm \Omega_{\text{eff}}(t)$ and are energetically only allowed for a phonon-photon or photon-photon transition. In a coherent process, the photons must appear in pairs of a low- and high-frequency photon (thus obeying the conservation of energy) and are the result of two-photon scattering from the laser field. In the absence of an asymmetric cavity or for a sufficiently large cavity mode, this leads to a symmetric spectral response.

On the other hand, incoherent interactions involving the phononic environment facilitate also single-photon transitions at those frequencies (more accurately, single-phonon single-photon transitions).

However, depending on the sign of the laser–QD detuning $\delta\omega_L > 0$ ($\delta\omega_L < 0$), the emission (absorption) of a phonon is required, and therefore only one of the transitions is efficiently supported.

In Fig. S7, we present the spectra obtained from our QD, where the laser is either blue- or red-detuned and its power is scanned. The scattered laser is filtered with a VBG. For $\delta\omega_L/2\pi = 131$ GHz (Fig. S7a), a low-frequency side peak is clearly visible, and its separation increases with the laser field. The absence of any emission at $\omega_L + \Omega_{\text{eff}}(t)$, is also a strong indication toward the single-photon transition. On the other hand, if $\delta\omega_L/2\pi = -131$ GHz (Fig. S7b), we identify two side peaks at $\omega_L \pm \Omega_{\text{eff}}(t)$, which are much weaker in intensity than in Fig. S7a. Since at $T = 4$ K the phonon occupation is low, the incoherent, phonon-assisted single-photon transition becomes significantly less efficient for a red-detuned laser pulse and symmetric two-photon scattering dominates.

Combining these findings, we conclude that the low-frequency side peak investigated in the Main Text for $\delta\omega_L > 0$ is (almost exclusively) a result of phonon-assisted emission.

In addition to the laser power scan presented in the Main Text, Fig. S8 shows how the two-photon spectrum changes when $\delta\omega_L$ is varied at a constant power of 250 nW. As expected from dressed-state emission (see Eq. 2 in the Main Text), a smaller laser–QD detuning induces a stronger level splitting, and thus a larger frequency shift of the 1st photon, that is, the side peak.

Finally, Fig. S9 reproduces the data from Fig. 4a of the Main Text but with a rescaled time axis to include the uncorrelated side peaks. The grey areas indicate the integration intervals that are used to calculate the $g^{(2)}(0)$ values and include more than 99.9% of the uncorrelated coincidences. As discussed in the Methods, the additional features around ± 5 ns are attributed to optical reflections in the setup.

Supplementary Note 6. FREQUENCY-FILTERED TIME TRACES

The dressed-state model suggests that the level splitting, and thus the emission frequency, changes throughout the laser pulse interaction, as captured in

$$\omega_{\text{QD}}(t) = \omega_L - \Omega_{\text{eff}}(t) \ , \tag{13}$$

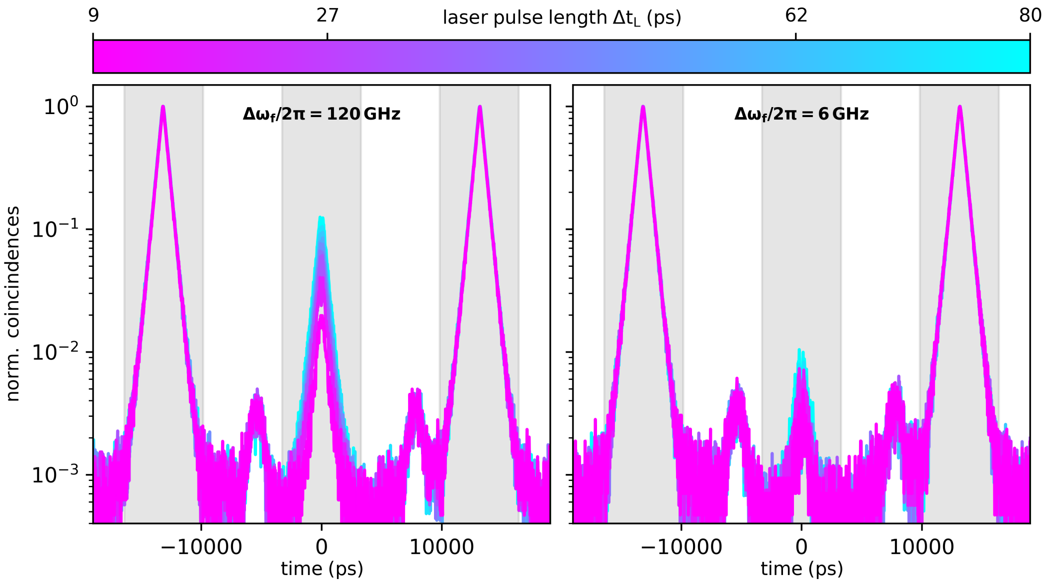

Supplementary Figure 9. **Autocorrelation of (un-)filtered QD emission.** For a constant laser power of 185 nW, the pulse length is varied and the second-order autocorrelation $g^{(2)}(\tau)$ is measured for each step using a (120 ± 1) GHz VBG filter (left panel) and a (6.0 ± 0.3) GHz etalon (right panel). The grey areas indicate the 6.58 ns long integration intervals used for the $g^{(2)}(0)$ estimation.

where $\Omega_{\mathrm{eff}}(t) = \sqrt{\Omega^2(t) + \delta\omega_L^2}$ is the effective Rabi frequency. Using the setup depicted in Fig. S2 without the fixed etalon but including the scanning etalons, we can measure the temporal response for different filter frequencies ω_{f}. Simply correlating the optical signal of the filtered HBT path with the laser clock yields a time trace for each specific filter frequency $\delta\omega_{\mathrm{f}} = \omega_{\mathrm{f}} - \omega_0$ as depicted in Fig. S10. Throughout the panels, the central frequency of the filter is shifted stepwise to larger detunings as indicated by the color bar.

Initially, when the filter overlaps well with ω_0, we find an early rise in signal counts at the very beginning of the laser pulse (indicated in gray) that plateaus first (or even drops slightly) and is then followed by a second much stronger signal increase and a slow exponential decay. This behavior starts to change both qualitatively and quantitatively, when red-detuning the filter from ω_0. The early rise lasts longer, reaching a higher signal level, and the following drop in counts in more pronounced. The second rising-edge peaks earlier, so that the maximum signal strength reduces and, more importantly, the falling edge becomes more Gaussian (see e.g. darkest curve in the second panel). This trend continues as the red-shift enlarges and the signal drop after the first rise slowly washes out until there is only a single smooth rise and fall in detected counts (see curve with highest peak in the fourth panel). Also note that the global peak height increases again throughout the fourth panel. In the last panel, the shape of the signal does not change anymore as it steadily declines in signal strength.

Recalling that a Gaussian laser pulse causes the QD transition frequency to first decrease until the pulse peaks and then increase again (see Eq. (13)), the main features of our findings are readily explained. For most filter positions, there are two points in time when they overlap with the time-varying emission frequency; once during the rising edge of the laser field and a second time when the field strength subsides. Only the maximum frequency shift occurs for just a single point in time, namely when the laser pulse peaks.

This behavior is captured in Fig. S10 where for all filter settings $\delta\omega_{\mathrm{f}} < \delta\omega_{\mathrm{max}}$ (for these laser settings, $\delta\omega_{\mathrm{max}}/2\pi \approx -45$ GHz, see Fig. S8), two peaks appear in the time trace. The temporal separation of these peaks shrinks with increasing $\delta\omega_{\mathrm{f}}$ until they merge into one at $\delta\omega_{\mathrm{f}} = \delta\omega_{\mathrm{max}}$. Additionally, we clearly see that photons near ω_0 exhibit an exponential decay as expected from spontaneous emission, whereas photons at significantly red-shifted frequencies must be emitted while the laser pulse is present and therefore inherit the temporal shape of the laser pulse.

To analyze and understand the process quantitatively, one must also incorporate the time-dependent probability of the emitter being in the excited state. For phonon-assisted excitation, involving non-Markovian processes, this is commonly done using numerical methods, including time–path integrals[14]. However, some intuition about the excitation probability can be gained from considering the phonon spectral density $J(\omega)$. As indicated in Supplementary Note 1, there is a Rabi frequency Ω_{eff} that maximizes the phonon coupling and overshooting causes less efficient

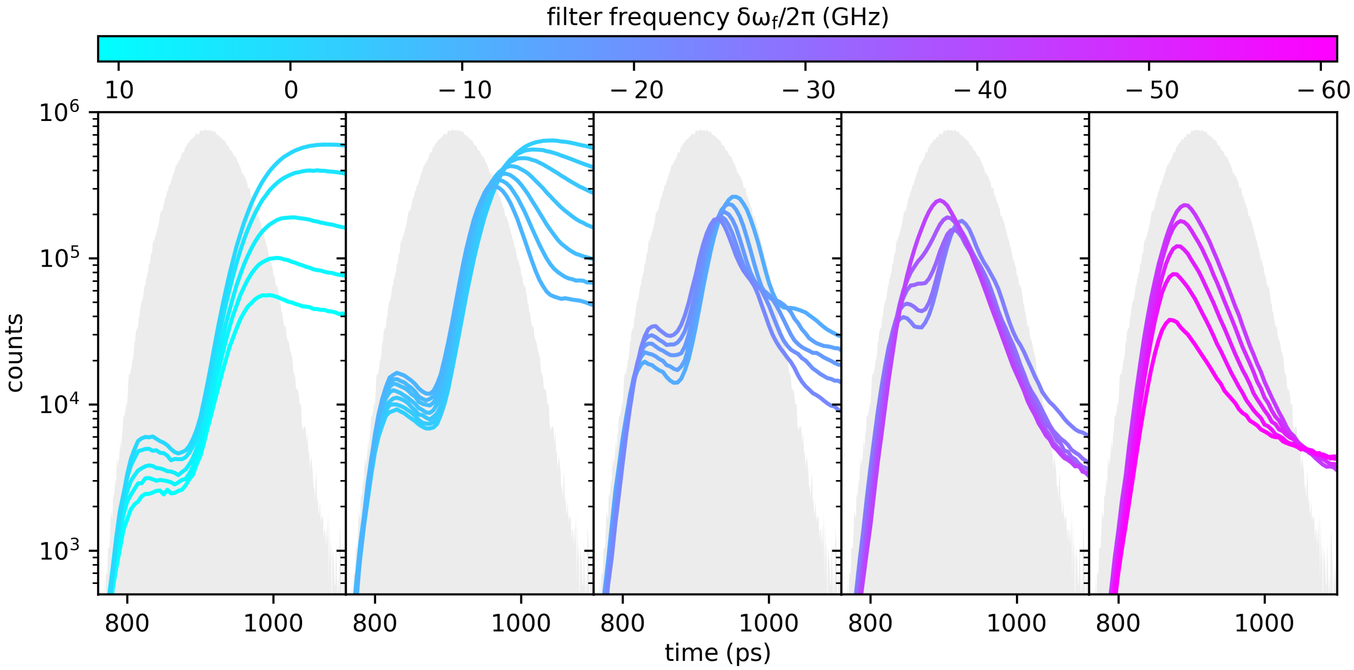

Supplementary Figure 10. **Histogram of frequency filtered QD emission.** For (80 ± 1) ps long laser pulses with 250 nW and $\delta\omega_L/2\pi = 125$ GHz, the QD emission is filtered using the set of scanning etalons shown in Fig. 2. The filtered channel is correlated with the laser clock to construct the histograms for every filter position $\delta\omega_\mathrm{f} = \omega_\mathrm{f} - \omega_0$. The curves are colored according to $\delta\omega_\mathrm{f}$ as indicated by the color bar. Due to the Fourier-transform limit, the original temporal signal is exponentially broadened by the (5.3 ± 0.3) GHz wide Lorentzian filter. The errors were calculated using Poissonian statistics and are too small to be visible.

phonon coupling, thus less efficient population inversion of the QD. This non-monotonic evolution of the phonon coupling throughout the laser pulse can lead to strong phonon interactions at the beginning and end of a laser pulse but weak interaction at its peak field amplitude. The reduced phonon coupling at large effective Rabi frequencies was recently also shown to revive the Rabi oscillations of a coherently driven QD[15]. While it is necessary to incorporate these effects to model the (frequency-filtered) emission probability across large parameter ranges, we note that the detuning and driving field used in Fig. S10 are small enough such that $\Omega_\mathrm{eff}(t)$ never exceeds the optimal phonon coupling. Therefore, the time-dependent phonon scattering rate does not feature an inflection point and the dip (or kink) seen in Fig. S10 can be attributed solely to frequency-selective detection.

Supplementary References

1. B. R. Mollow, Power spectrum of light scattered by two-level systems, Physical Review Letters **188** (1969).
2. B. Krummheuer, V. M. Axt, and T. Kuhn, Theory of pure dephasing and the resulting absorption line shape in semiconductor quantum dots, Physical Review B **65**, 10.1103/PhysRevB.65.195313 (2002).
3. T. E. Hodgson, L. Viola, and I. D'Amico, Decoherence-protected storage of exciton qubits through ultrafast multipulse control, Physical Review B **78**, 10.1103/PhysRevB.78.165311 (2008).
4. J. H. Quilter, A. J. Brash, F. Liu, M. Glässl, A. M. Barth, V. M. Axt, A. J. Ramsay, M. S. Skolncik, and A. M. Fox, Phonon-assisted population inversion of a single ingaas/gaas quantum dot by pulsed laser excitation, Phys. Rev. Lett. **114** (2015).
5. A. M. Barth, S. Lüker, A. Vagov, D. E. Reiter, T. Kuhn, and V. M. Axt, Fast and selective phonon-assisted state preparation of a quantum dot by adiabatic undressing, Phys. Rev. B **94**, 045306 (2016).
6. S. Lüker and D. E. Reiter, A review on optical excitation of semiconductor quantum dots under the influence of phonons, Semiconductor Science and Technology **34**, 063002 (2019).
7. D. Reiter, S. Lüker, K. Gawarecki, A. Grodecka-Grad, P. Machnikowski, V. Axt, and T. Kuhn, Phonon effects on population inversion in quantum dots: Resonant, detuned and frequency-swept excitations, Acta Physica Polonica A **122**, 1065–1068 (2012).

8. C. Gustin and S. Hughes, Efficient pulse–excitation techniques for single photon sources from quantum dots in optical cavities, Advanced Quantum Technologies **3**, 10.1002/qute.201900073 (2020).
9. M. Cosacchi, F. Ungar, M. Cygorek, A. Vagov, and V. M. Axt, Emission-frequency separated high quality single-photon sources enabled by phonons, Phys. Rev. Lett. **123**, 017403 (2019).
10. M. Vyvlecka, L. Jehle, C. Nawrath, F. Giorgino, M. Bozzio, R. Sittig, M. Jetter, S. L. Portalupi, P. Michler, and P. Walther, Robust excitation of C-band quantum dots for quantum communication, Appl. Phys. Lett. **123**, 174001 (2023).
11. K. Boos, S. K. Kim, T. Bracht, F. Sbresny, J. M. Kaspari, M. Cygorek, H. Riedl, F. W. Bopp, W. Rauhaus, C. Calcagno, J. J. Finley, D. E. Reiter, and K. Müller, Signatures of dynamically dressed states, Phys. Rev. Lett. **132**, 053602 (2024).
12. S. Liu, C. Gustin, H. Liu, X. Li, Y. Yu, H. Ni, Z. Niu, S. Hughes, X. Wang, and J. Liu, Dynamic resonance fluorescence in solid-state cavity quantum electrodynamics, Nature Photonics **18**, 318 (2024).
13. J. Cohen-Tannoudji, C. amd Dupont-Roc and G. Grynberg, The dressed atom approach, in *Atom—Photon Interactions* (John Wiley & Sons, Ltd, 1998) Chap. 6, pp. 407–514.
14. A. Vagov, M. D. Croitoru, M. Glässl, V. M. Axt, and T. Kuhn, Real-time path integrals for quantum dots: Quantum dissipative dynamics with superohmic environment coupling, Phys. Rev. B **83** (2011).
15. L. Hanschke, T. K. Bracht, E. Schöll, D. Bauch, E. Berger, P. Kallert, M. Peter, A. J. Garcia, S. F. Covre da Silva, S. Manna, A. Rastelli, S. Schumacher, D. E. Reiter, and K. D. Jöns, Experimental measurement of the reappearance of rabi rotations in semiconductor quantum dots, Physical Review Letters **135**, 10.1103/s212-43gs (2025).
