# Supplementary material for: Asymmetric two-photon response of an incoherently driven quantum emitter
Source: Nat Commun. 2026 May 9;17:6400. doi: 10.1038/s41467-026-72706-z (PMC13376212; doi:10.1038/s41467-026-72706-z)
Supplement: Supplementary file 2 — Transparent Peer Review file [file 41467_2026_72706_MOESM2_ESM.pdf]

nature portfolio

Peer Review File

Asymmetric two-photon response of an incoherently driven quantum emitter

Corresponding Author: Mr Lennart Jehle

This file contains all reviewer reports in order by version, followed by all author rebuttals in order by version.

Version 0:

Reviewer comments:

Reviewer #1

(Remarks to the Author)
The manuscript by Jehle et al. analyzes the re-excitation of a quantum emitter (a quantum dot, QD, in a cavity) driven incoherently by longitudinal acoustic (LA) phonon-assisted pulsed laser excitation. Re-excitation causes the consecutive emission of two photons within a single laser pulse period, thereby limiting the purity of the source, which is a crucial performance metric for single photon sources for photonic quantum technologies.
LA-phonon excitation is very promising since it allows separation of pump and single photon signals simply by spectral filtering, in contrast with resonant excitation schemes that require spatial mode filtering. While LA-phonon excitation has been explored in the past for demonstrating high purity, bright QD single photon sources, re-excitation has not yet received much attention - contrasting with the case of strictly resonant excitation.
The manuscript brings to light many new compelling insights into the phenomenon. First, signatures of the temporal ordering of the two emitted photons are presented via a clever HBT correlation measurement and post-selection of early versus later (time-tagged) detection events. Spectrally resolved correlation measurements then are used to show that the first QD-emitted photon undergoes a spectral shift, resulting in a spectral asymmetry that contrasts with that observed under resonant driving. A linear evolution of the spectral shift with respect to the pump field amplitude evidences the dynamic Stark effect. The authors show preservation of single-photon purity as a function of excitation pulse length, upon application of an appropriate filter that spectrally excludes the second photon.
The supplementary information also shows frequency-filtered time traces of the QD emission, which provides experimental evidence for the time-dependent level splitting predicted by the dynamic Stark effect during phonon-assisted re-excitation. This is a very compelling result that points to potential new possibilities for exploring engineered phononic density of states.
The article is technically solid and certainly there is enough novelty and appeal for researchers working with III-V epitaxial quantum dots as single-photon emitters. The subject is somewhat specialized for a broader readership, however with some small adjustment it may be made to appeal more broadly.

I have a few comments and suggestions to provide the reader a more complete picture of the subject, before the manuscript can be acceptable for publication.

1 - The subject is somewhat specialized for a broader readership. It could be worthwhile expanding a bit on the generality of the re-excitation issue and potential for phonon-assisted excitation in other solid-state quantum emitter systems.

2 - Fig. 3a suggests some reasonable spectral overlap between the 1st and 2nd photons. Upon spectral filtering, I would expect some overall source efficiency degradation. Can the authors quantify such degradation as a function of the drive power, and what drive powers would be optimal or maximal (e.g. beyond which the technical becomes impractical)? At least a comment would be desirable here about trade-offs between spectral shifts / filtering and excitation probability, towards optimal efficiency. The latter is an important performance metric for single-photon sources that is not extensively discussed in the text.

3 - In Fig. 3b, is there any adjustment that is done in the horizontal axis for each scan, to ensure that the taller, 2nd photon peak is always at \Delta\omega = 0 ? In other words, how do the authors ensure the repeatability of the piezo scanner filters spectral location?

4 - In the measurements discussed in Supplementary Note 4, the arguments provided to explain the data are reasonable,

however measuring at different detunings could be helpful to complete the picture. In particular, it is a bit puzzling that the laser peak maximum corresponds to an emission minimum - I guess this means that the laser is initially tuned to the maximum overall excitation efficiency, but the optimal effective QD Rabi frequency is actually not achieved at the pulse maximum. Is that right? So it is reasonable to think that detuning the laser could potentially make the laser peak coincide with the optimal QD spectral detuning. Have the authors measured the excitation laser frequency dependency of the phenomenon?

Reviewer #2

(Remarks to the Author)
The authors report a detailed investigation of the asymmetric two-photon spectrum originating from blue-detuned laser excitation. The authors use In(Ga)As quantum dot in a CBG cavity, which have been shown to be a bright, indistinguishable C-band single photon emitter in prior work. The primary advance of the present paper is the identification of sequentially emitted two photons in both the time and frequency domains, by their advanced multichannel time-correlated spectral measurement technique. Based on these results, the authors reveal the temporal and spectral asymmetry of the two photons. Furthermore, by applying narrow-band filtering, high single-photon purity and low multi-photon contribution can be recovered even under relatively long excitation pulses, which is important for developing high-purity, Purcell-enhanced quantum light sources.

Overall, the manuscript is clearly written, the experimental results are solid, and the conclusions are convincing. Before publication, I have a few suggestions as minor comments:

1. Although the authors have shown detailed g2(0) data, presenting the multi-photon probabilities (with and without spectral filtering) under different laser pulse durations would help readers gain a more intuitive understanding of the effects.

2. The introduction cites Ref. 37, which reports a Purcell factor of 25; however, this is not the highest value currently achieved in QD–cavity coupling systems. The authors should include references reporting higher Purcell factors.

3. Is spectral filtering to suppress multi-photon contributions unique to phonon-assisted excitation, or could similar improvement also be achieved under resonant excitation? Some comments about this should be given.

4. Adding some numerical simulations to fit or support the experimental data would be helpful, especially for the asymmetric two-photon spectrum.

5. Citations to the Supplementary Information should refer to specific sections or figure numbers to help readers accurately locate the corresponding content.

6. In Fig. 4a, the side peaks used for g2 normalization should also be shown.

7. In Supplementary Fig. 4, the tick values of the color bar should be included.

8. In Supplementary information, line 72, “conservation of energy” typo.

Reviewer #3

(Remarks to the Author)

The authors investigate re-excitation dynamics under longitudinal-acoustic (LA) phonon–assisted pumping of a quantum dot, providing a time-resolved separation of the first and second photons originating from multiphoton emission processes. The work identifies clear spectral signatures of re-excitation, including a dressed-state–induced red shift of the first photon. The authors further propose a method to extract the Rabi frequency under incoherent excitation conditions and demonstrate a practical scheme for suppressing multiphoton noise using frequency filtering. Overall, the topic is timely and highly relevant to solid-state quantum optics and the development of quantum-dot–based single-photon sources.

I find the experimental characterization of multiphoton re-excitation under phonon-assisted pumping particularly interesting and potentially among the first demonstrations of its kind, as claimed by the authors. The work convincingly demonstrates the temporal ordering of photon emission using combined time and frequency resolution. This provides a practical and experimentally accessible approach for reducing multiphoton noise without the need for ultrafast electro-optic modulation, which is a significant advantage.

However, I feel that the experimental interpretation relies heavily on simplifying assumptions regarding the dressed-state picture. As a result, some readers may find the theoretical treatment insufficient. At a minimum, more detailed theoretical discussion should be provided in the Supplementary Material. In particular, the following points should be addressed:

1. The dressed states (|\alpha, N\rangle) and (|\beta, N\rangle) are introduced, but the physical meaning of (\alpha) and (\beta) remains insufficiently specified. The authors argue that when the dressed-state splitting (\Omega_eff(t)) matches the

phonon spectral density, phonon emission leads to population inversion. However, the manuscript does not adequately discuss the theoretical modeling of the phonon spectral density, relying mainly on references to earlier work. It appears that by “matching,” the authors refer to the frequency at which the phonon spectral density is maximal. Importantly, this condition can, in principle, be satisfied for both positive and negative laser detunings (\delta\omega_L). Nevertheless, previous studies (e.g., Ref. [28]) have shown that phonon-assisted inversion depends strongly on the sign of the detuning. Furthermore, dressed-state populations themselves depend on the detuning sign due to asymmetric phonon emission and absorption processes. These aspects should be discussed more clearly.

2. The phonon-assisted excitation mechanism presented in the manuscript appears to operate primarily in the far off-resonant driving regime. This limitation should be explicitly stated and discussed, as it is important for assessing the general applicability of the proposed scheme.

3. The authors state that the cavity influence is negligible in the reported experiments. While this may be reasonable, a more quantitative analysis or estimate (e.g., comparison of relevant rates or coupling strengths) would significantly strengthen this claim.

4. The procedure used to define and separate the “first” and “second” photons through correlation gating requires clearer justification. In particular, a discussion of possible systematic biases introduced by this method, and how they may affect the extracted results, would improve the rigor of the analysis.

5. A brief discussion of how sensitive the re-excitation dynamics and phonon-assisted processes are to temperature would be valuable, especially for practical implementations.

Overall, I find the manuscript suitable for publication after the above minor revisions are addressed.

Version 1:

Reviewer comments:

Reviewer #1

(Remarks to the Author)
I find the responses to my comments generally satisfying and the changes to the text and SI sufficient for me to recommend publication in Nature Communications without further changes. I appreciate in particular the inclusion of the supplemental section on dressed state physics and the clarification about my question regarding the filtered emission. I think the authors have also sufficiently broadened the scope of the technique through the color center citations and comments.

Reviewer #2

(Remarks to the Author)
The authors have responded to all referee comments and have performed additional theoretical analysis. All issues raised in the previous review have been adequately addressed, and the manuscript has been strengthened accordingly. I am satisfied with the revisions and recommend publication in Nature Communications.

Reviewer #3

(Remarks to the Author)
The authors have carefully addressed all comments raised by the reviewers. The revised manuscript incorporates the requested clarifications and additional references, and the supplementary material has been expanded to provide a clearer theoretical description of the dressed-state picture and phonon-assisted excitation processes.
In my view, the authors have satisfactorily addressed the reviewers’ comments, and the manuscript is suitable for publication in its revised form.

Open Access This Peer Review File is licensed under a Creative Commons Attribution 4.0 International License, which permits use, sharing, adaptation, distribution and reproduction in any medium or format, as long as you give appropriate credit to the original author(s) and the source, provide a link to the Creative Commons license, and indicate if changes were made.
In cases where reviewers are anonymous, credit should be given to 'Anonymous Referee' and the source.
The images or other third party material in this Peer Review File are included in the article's Creative Commons license, unless indicated otherwise in a credit line to the material. If material is not included in the article's Creative Commons license and your intended use is not permitted by statutory regulation or exceeds the permitted use, you will need to obtain permission directly from the copyright holder.
To view a copy of this license, visit https://creativecommons.org/licenses/by/4.0/

Point-by-point replies

We thank the Reviewers for their helpful feedback, and we hereby provide a point-by-point reply to each of their comments.

. . . .

1. In response to Reviewer 1:

> **Reviewer 1:**
> The manuscript by Jehle et al. analyzes the re-excitation of a quantum emitter (a quantum dot, QD, in a cavity) driven incoherently by longitudinal acoustic (LA) phonon-assisted pulsed laser excitation. Re-excitation causes the consecutive emission of two photons within a single laser pulse period, thereby limiting the purity of the source, which is a crucial performance metric for single photon sources for photonic quantum technologies. LA-phonon excitation is very promising since it allows separation of pump and single photon signals simply by spectral filtering, in contrast with resonant excitation schemes that require spatial mode filtering. While LA-phonon excitation has been explored in the past for demonstrating high purity, bright QD single photon sources, re-excitation has not yet received much attention - contrasting with the case of strictly resonant excitation. The manuscript brings to light many new compelling insights into the phenomenon. First, signatures of the temporal ordering of the two emitted photons are presented via a clever HBT correlation measurement and post-selection of early versus later (time-tagged) detection events. Spectrally resolved correlation measurements then are used to show that the first QD-emitted photon undergoes a spectral shift, resulting in a spectral asymmetry that contrasts with that observed under resonant driving. A linear evolution of the spectral shift with respect to the pump field amplitude evidences the dynamic Stark effect. The authors show preservation of single-photon purity as a function of excitation pulse length, upon application of an appropriate filter that spectrally excludes the second photon. The supplementary information also shows frequency-filtered time traces of the QD emission, which provides experimental evidence for the time-dependent level splitting predicted by the dynamic Stark effect during phonon-assisted re-excitation. This is a very compelling result that points to potential new possibilities for exploring engineered phononic density of states. The article is technically solid and certainly there is enough novelty and appeal for researchers working with III-V epitaxial quantum dots as single-photon emitters. The subject is somewhat specialized for a broader readership, however with some small adjustment it may be made to appeal more broadly.
> I have a few comments and suggestions to provide the reader a more complete picture of the subject, before the manuscript can be acceptable for publication.

Our reply: We thank the Reviewer for their very positive evaluation of our work and their constructive feedback that helped to improve the quality of the manuscript.
In the replies below, we have addressed their points and implemented the suggested changes in the Main Text and Supplementary Information.

1.1. Reviewer 1:
The subject is somewhat specialized for a broader readership. It could be worthwhile expanding a bit on the generality of the re-excitation issue and potential for phonon-assisted excitation in other solid-state quantum emitter systems.

Our reply:
We greatly appreciate the suggestions and agree that the impact of our findings extends to other solid-state quantum emitters–especially those for which phonon-assisted excitation schemes were already demonstrated–and that this fact could have been highlighted more explicitly. To this end, we now cite four works on color centers in hBN and TMDs that employ exciton-phonon coupling in their driving schemes. Specifically, Refs. 35 and 36 also use LA phonon interactions, indicating that the underlying physics in these systems and QDs is very similar. In the Discussion, we revisit the argument and note that (some of) the results should be transferable.

Regarding the generality, we feel that both the abstract and the third paragraph of the introduction convey the fundamental nature of the re-excitation phenomenon and the extent of the resulting challenge for single-photon generation using quantum emitters. Foreboding that the problem's scale will only increase as Purcell enhancement improves underscores the challenge's importance and urgency.
The Discussion, on the other hand, was revised to better reflect the breadth of the phenomenon (both in terms of the type of quantum emitter and the driving scheme). The Reviewer's comment also prompted us to encourage additional research on other excitation methods to complete the picture. Here, we identified both adiabatic rapid passage and the so-called SUPER scheme as promising candidates for greater resilience against multiphoton re-excitation.

1.2. Reviewer 1:
Fig. 3a suggests some reasonable spectral overlap between the 1st and 2nd photons. Upon spectral filtering, I would expect some overall source efficiency degradation. Can the authors quantify such degradation as a function of the drive power, and what drive powers would be optimal or maximal (e.g. beyond which the technical becomes impractical)? At least a comment would be desirable here about trade-offs between spectral shifts / filtering and excitation probability, towards optimal efficiency. The latter is an important performance metric for single-photon sources that is not extensively discussed in the text.

Our reply:
We thank the Reviewer for raising an important point. When working with single-photon sources, one seldom optimizes for a single figure of merit but often seeks to enhance two, three, or even more simultaneously. Typically, however, these are not (fully) independent, and once one metric starts to decrease while another is still increasing, one must choose how to balance between them; this presents the first challenge when aiming for a joint optimization (see Ref. 29 (31) [from here on, all references in brackets refer to the revised version and those without to the submitted version]). The second is simply the size of the parameter space. Here, it includes not only the pump laser parameters – power, detuning, and pulse length – but also the filter's spectral shape and width, the

spectral density of the QD-phonon coupling, and the cavity mode and QD lifetime.

Returning to the specific question of the Reviewer, assuming all parameters except the laser power are fixed, we have two regimes. While the effective Rabi frequency induced by the driving field is smaller than the one achieving maximal phonon coupling (that is, $J(\Omega_{\text{eff}}) < \max_\omega J(\omega)$), increasing the driving field enhances the excitation probability and enlarges the spectral shift. Thus, increasing the laser field simultaneously improves generation efficiency and filtering. However, once the driving field causes Ω_{eff} to exceed the maximal phonon coupling frequency, the excitation probability slowly decreases, whereas the Stark shift continues to increase. How to weigh the single-photon generation efficiency against the multiphoton probability (p_{m}) when optimizing? As shown in our previous work (Ref. 29 (31)), one must choose a metric that combines the two into a single figure of merit, and often, this metric is derived from the application. However, fixing the application limits the transferability of the results, so one may prefer to report the photon-number probabilities instead, allowing others to optimize based on their application.
Still, for orientation, we can provide some overview of the scalings. The pump parameters that optimize the generation efficiency for this QD ($\approx 250\,\text{nW}$ laser power at $\delta\omega_L/2\pi \approx 120\,\text{GHz}$ and $\Delta t_L \approx 20\,\text{ps}$) already induce a significant Stark shift. Using the same detuning and laser power but four times longer pulses (Fig. 3b) lowers the maximum effective Rabi frequency by a factor of 2, but the spectral shift (measured at the intensity peak) is still >40 GHz or >320 pm. Thus, assuming single-photon emission with a FWHM of ≈1 GHz as commonly found for Fourier-transform-limited QDs, even "normal" laser parameters (i.e., not explicitly optimized for the spectral filtering) achieve a small spectral overlap between the 1st and 2nd photons, such that additional optimization may only provide small improvements.
The exact nature of the trade-off between p_1 and p_{m} depends on the filter, the specific QD, and many additional parameters as mentioned above, making further generalization difficult. While we believe that one can identify more and less relevant regions in this parameter space, quantifying the trade-offs among the various parameters requires substantial experimental effort or a full theoretical simulation. The focus of this work, on the other hand, is to report a phenomenon not previously observed in phonon-assisted excitation, demonstrating good qualitative and quantitative agreement with the dressed-state model through selected parameter scans.

In response to the Reviewer's comment, we have included a stripped-down version of this answer at the end of the "Restoring high single-photon purities" section (starting at ln. 322), which we hope conveys the central message. We also added the optimal pump parameter for a short pulse to the Methods.

1.3. Reviewer 1:

In Fig. 3b, is there any adjustment that is done in the horizontal axis for each scan, to ensure that the taller, 2nd photon peak is always at $\Delta\omega = 0$? In other words, how do the authors ensure the repeatability of the piezo scanner filters spectral location?

Our reply:
The frequency-resolved data is acquired by stepwise scanning the angle between the beam path and the etalon's surface normal. To this end, each etalon is mounted on a high-precision closed-loop piezo-actuated rotation stage (*attocube ECR5050hs/Al/RT*). Using

internal position feedback, the etalons' transmission wavelengths are then tuned by scanning over a predefined range of angles rather than piezo voltages. The conversion from an angle to a wavelength (or frequency) dependence is performed based on a calibration table previously recorded using a cw laser with a linewidth of 20 kHz. Hence, no correction or other post-processing was applied to the raw data to align the tall peaks at $\Delta\omega = 0$ across scans with different laser powers, but the voltage-to-angle and angle-to-wavelength transformations were set a priori.
In other words, repeatability is ensured solely by the piezo stage's internal feedback control, which, according to the manufacturer, is in the microdegree range. In the relevant angle range, the angle tunability of the etalons used here may be linearized, yielding approximately 125 GHz/° and 180 GHz/°, corresponding to an overall repeatability of less than a few MHz.

We thank the Reviewer for this question, which prompted us to update Supplementary Note 1 (2) to include these details.

> **1.4. Reviewer 1:**
> In the measurements discussed in Supplementary Note 4, the arguments provided to explain the data are reasonable, however measuring at different detunings could be helpful to complete the picture. In particular, it is a bit puzzling that the laser peak maximum corresponds to an emission minimum - I guess this means that the laser is initially tuned to the maximum overall excitation efficiency, but the optimal effective QD Rabi frequency is actually not achieved at the pulse maximum. Is that right? So it is reasonable to think that detuning the laser could potentially make the laser peak coincide with the optimal QD spectral detuning. Have the authors measured the excitation laser frequency dependency of the phenomenon?

Our reply:
We thank the Reviewer for the question, which gives us the opportunity to clarify the measurement described in Supplementary Note 4 (Note 6).
The motivation of Fig. S6 (S9) is to explicitly demonstrate the dynamical spectral shift of the emission wavelength throughout the interaction with the laser pulse. To this end, the QD emission is passed through the cascaded etalons with an approximate Lorentzian FWHM of 5.3(3) GHz before being detected by an SNSPD and correlated with the laser clock to create the histograms. The full signal stream is analyzed (no postselection on two-photon events). Hence, the measurement shows the temporal evolution of the detected count rate across different spectral slices as indicated by the color of each curve. The laser settings are constant throughout the measurement.
At the peak laser intensity, the effective Rabi frequency and thus the Stark shift are maximal, causing the emission at $\omega_{QD}(t) = \omega_L - \Omega_{\text{eff}}(t)$ to be far off the undisturbed frequency ω_0. Since the curves in the left panels correspond to $\delta\omega_f/2\pi \approx 0$ (that is, the filter transmits only light close to the undisturbed frequency ω_0), the emission probability dips around the laser peak. At this point in time, the QD emits at $\omega_0 - \max_t \Omega_{\text{eff}}(t)$ such that only a little light still passes through the filter. On the other hand, when the filter offset is $\delta\omega_f/2\pi \approx 45$ GHz (right panels), the curves reach their maxima near the laser's peak intensity.

We have measured the two-photon spectrum for three different laser-QD detunings $\delta\omega_L$ (Fig. S5 (S8)) and find that the maximum Stark shift reduces for larger detuning, just as predicted by the dressed state model. The farther the laser is from the resonance

frequency, the smaller the Stark effect (also captured in the above Eq. 1 of the Main Text). However, we do not expect any qualitative change in the temporal structure (i.e., the measurement type shown in S6 (S9)) for a different laser-QD detuning $\delta\omega_L$. At the peak laser intensity, the probability of detecting a photon with frequency ω_0 remains minimal.

We infer from the Reviewer's question that they expect a greater impact of the time-dependent phonon scattering rate on this measurement. However, because of the long laser pulses, the effective Rabi frequency does not exceed the frequency of the optimal phonon coupling. The laser power and detuning used for most measurements of this work ($\approx$250 nW and $\delta\omega_L \approx 120$ GHz) were set to maximize the excitation probability for a $\approx$20 ps pulse, indicating that for those parameters $\max_t J(\Omega_{\mathrm{eff}}(t)) \approx \max_\omega J(\omega)$. Using the same laser power for a $\approx$80 ps pulse, the maximum laser field is only half as large. At the same time, the interaction time is four times longer, such that a high excitation probability is achieved even though the phonon scattering rate is lower.

A distinct feature in the time trace of the emission probability (i.e., a kink or even a dip), which is solely caused by varying phonon coupling, is only expected if $\max_t \Omega_{\mathrm{eff}}(t)$ well exceeds $\max_\omega J(\omega)$. The results shown in Fig. S6 (S9) are thus fully attributed to the narrow spectral filtering.

We agree that introducing the subtleties of time-dependent phonon coupling in this context (the last paragraph of Supplementary Note 4 (6)) was misleading. As we have added another Supplementary Note (Note 1) detailing the dressed-state picture, we have moved the discussion of the phonon scattering rate and adjusted Supplementary Note 4 (6) accordingly. We believe that the revised version is more clearly structured and hope it prevents any misunderstanding.

....

2. In response to Reviewer 2:

Reviewer 2:
The authors report a detailed investigation of the asymmetric two-photon spectrum originating from blue-detuned laser excitation. The authors use In(Ga)As quantum dot in a CBG cavity, which have been shown to be a bright, indistinguishable C-band single photon emitter in prior work. The primary advance of the present paper is the identification of sequentially emitted two photons in both the time and frequency domains, by their advanced multichannel time-correlated spectral measurement technique. Based on these results, the authors reveal the temporal and spectral asymmetry of the two photons. Furthermore, by applying narrow-band filtering, high single-photon purity and low multi-photon contribution can be recovered even under relatively long excitation pulses, which is important for developing high-purity, Purcell-enhanced quantum light sources.
Overall, the manuscript is clearly written, the experimental results are solid, and the conclusions are convincing. Before publication, I have a few suggestions as minor comments.

Our reply:
We are pleased to learn that the Reviewer is convinced by our experimental data and their interpretation.
In the following answers, we address their helpful and constructive comments and questions and have implemented changes to the revised manuscript accordingly.

2.1. Reviewer 2:

Although the authors have shown detailed g2(0) data, presenting the multi-photon probabilities (with and without spectral filtering) under different laser pulse durations would help readers gain a more intuitive understanding of the effects.

Our reply:
We agree that the multiphoton component is highly relevant for applications. On the other hand, in the context of discussing the performance of single-photon sources, the $g^{(2)}(0)$ value is commonly used, mainly because it can be directly measured and is independent of the detected count rate.
To infer the multiphoton component

$$p_{\mathrm{m}} \leq \frac{1 - \eta_{\mathrm{sys}}\, g^{(2)}(0) - \sqrt{1 - 2\eta_{\mathrm{sys}}\, g^{(2)}(0)}}{g^{(2)}(0)} \tag{1}$$

from experimental data, one must make some (reasonable) assumptions about the single-photon source and reliably measure the system efficiency η_{sys} (for instance, see SI of Ref. 29 (31) [from here on, all references in brackets refer to the revised version and those without to the submitted version]). Hence, p_{m} has a larger measurement uncertainty and is prone to artifacts arising from fluctuations in η_{sys}.
In our experiment, the strongest influence is caused by the sweep of the laser pulse length itself. When stretching the laser pulses while keeping the laser power fixed, the maximum effective Rabi frequency decreases, but the interaction time for which notable phonon coupling is achieved increases. This causes a nontrivial variation in the excitation probability and, thus, in the system efficiency, which in turn affects p_{m}. Similarly,

artifacts are induced when the detection rate varies during the scan (for example, polarization drift causing a change in detection efficiency on SNSPDs, or a fiber connection being redone, etc.). Since these are, by nature, difficult to predict, they can hardly be correct for.
Importantly, the $g^{(2)}(0)$ value as a relative measure of single and multiphoton events is insensitive to variations of η_{sys}, providing a better comparability of the single-photon purity of the source throughout the scan. For those reasons, we believe that the $g^{(2)}(0)$ value is more suitable to highlight the effect of re-excitation and its dependence on the laser pulse length.

> **2.2. Reviewer 2:**
> The introduction cites Ref. 37, which reports a Purcell factor of 25; however, this is not the highest value currently achieved in QD–cavity coupling systems. The authors should include references reporting higher Purcell factors.

Our reply:
We highly appreciate the Reviewer's remark, which prompted us to include another work [Nature Nanotech 13, 835-840 (2018)] and update the corresponding sentence in the Main Text. Since this experiment uses a photonic crystal cavity, we retained the previous reference (based on a circular Bragg grating) as well to highlight that multiple cavity architectures have recently achieved very high Purcell factors.
If the Reviewer has yet another reference in mind, we are happy to include it if it adds more depth to the statement.

> **2.3. Reviewer 2:**
> Is spectral filtering to suppress multi-photon contributions unique to phonon-assisted excitation, or could similar improvement also be achieved under resonant excitation? Some comments about this should be given.

Our reply:
Spectral filtering is indeed possible for strictly resonant excitation, as shown for the first time very recently in a yet-unpublished article (Ref. 53 (62)). However, in the resonant case, there is no Stark-induced shift in the emission wavelength, so the filtering efficiency (that is, the trade-off between maintaining a high single-photon efficiency and reducing the multiphoton noise) is much lower than in the phonon-assisted scheme presented here. Since these results were uploaded to arXiv just a few days before our work and have not yet been peer-reviewed, we included only a brief comment in our Discussion, pointing to the highly related work but abstaining from discussing their results in more detail.
Nonetheless, we agree that our mention of the work on resonant excitation could have been more precise, and we revised the sentence in the updated Main Text.

> **2.4. Reviewer 2:**
> Adding some numerical simulations to fit or support the experimental data would be helpful, especially for the asymmetric two-photon spectrum.

Our reply:
We fully agree that numerical simulations will further deepen understanding by allowing us to explore the full parameter space beyond what is currently technically feasible and also simplify the otherwise experimentally tedious optimization to identify the ideal pump and filter settings for a given QD and application.
However, the purpose of this manuscript is to provide fundamentally new insight into the re-excitation phenomenon, while leaving detailed quantitative analysis using simulations for future work. We present the first experimental data on re-excitation under phonon-assisted driving, detail the temporal structure of two-photon emission, highlight significant differences compared to the resonant counterpart, and demonstrate to the community the potential these insights provide to improve the performance of quantum dot single-photon sources. To support our interpretation of the results, we explain the findings using the well-tested dressed-state picture. In full accordance with the model, we report a shift in emission wavelength that is proportional to the effective Rabi frequency (both using power tuning, Fig. 3b, and through variation of laser-QD detuning $\delta\omega_L$, Fig. S5 (S8)). Furthermore, in Fig. S6 (S9), we explicitly show how the QD's emission wavelength changes over time, following the instantaneous effective Rabi frequency induced by the driving laser. Given the qualitative and quantitative agreement between the experimental data and the existing theoretical model, we believe our results and interpretations are convincing without the additional support of numerical simulations.

> **2.5. Reviewer 2:**
> Citations to the Supplementary Information should refer to specific sections or figure numbers to help readers accurately locate the corresponding content.

Our reply:
We thank the Reviewer for the suggestion and share their view that the Reader's orientation within the work may be improved. Consequently, we followed their recommendation and included explicit section information in the references that point to the Supplementary Information.

> **2.6. Reviewer 2:**
> In Fig. 4a, the side peaks used for g2 normalization should also be shown.

Our reply:
We thank the Reviewer for raising the point, but we respectfully disagree. We are aware that the display style of Fig. 4a, without the normalized side peaks, may not be "standard", but we have deliberately chosen it to emphasize the evolution of the re-excitation signature throughout various laser pulse lengths, which is captured by the structure and height of the central peak alone. Since all uncorrelated side peaks are normalized to 1, the curves for different pulse lengths overlap and convey no additional information, only drawing visual focus away from the correlated central peak. Additionally, the rescaling of the axes necessary to accommodate both peaks would make it much harder to see the relevant details in the central peak.
Although the figure might appear less familiar to well-informed readers from the single-photon community, we are convinced that displaying the data in this way does not distort the results and enhances insight into the specific temporal structure of the two-photon component.

However, we understand that some Readers might prefer a more common layout. For this reason, we have included a figure showing the same data but in the format as requested by the Reviewer in the Supplementary Information (Fig. S9).

> **2.7. Reviewer 2:**
> In Supplementary Fig. 4, the tick values of the color bar should be included.

Our reply:
We thank the Reviewer for noticing, and are very sorry for the formatting issue that caused the label ticks to be clipped. We have corrected the figure so it displays them properly.

> **2.8. Reviewer 2:**
> In Supplementary information, line 72, "conservation of energy" typo.

Our reply:
We are grateful to the Reviewer for pointing out the typo and have corrected it.

. . . .

3. In response to Reviewer 3:

> **Reviewer 3:**
> The authors investigate re-excitation dynamics under longitudinal-acoustic (LA) phonon–assisted pumping of a quantum dot, providing a time-resolved separation of the first and second photons originating from multiphoton emission processes. The work identifies clear spectral signatures of re-excitation, including a dressed-state–induced red shift of the first photon. The authors further propose a method to extract the Rabi frequency under incoherent excitation conditions and demonstrate a practical scheme for suppressing multiphoton noise using frequency filtering. Overall, the topic is timely and highly relevant to solid-state quantum optics and the development of quantum-dot–based single-photon sources.
> I find the experimental characterization of multiphoton re-excitation under phonon-assisted pumping particularly interesting and potentially among the first demonstrations of its kind, as claimed by the authors. The work convincingly demonstrates the temporal ordering of photon emission using combined time and frequency resolution. This provides a practical and experimentally accessible approach for reducing multiphoton noise without the need for ultrafast electro-optic modulation, which is a significant advantage.
> However, I feel that the experimental interpretation relies heavily on simplifying assumptions regarding the dressed-state picture. As a result, some readers may find the theoretical treatment insufficient. At a minimum, more detailed theoretical discussion should be provided in the Supplementary Material. Overall, I find the manuscript suitable for publication after the minor revisions are addressed. In particular, the following points should be addressed.

Our reply:
We greatly appreciate the Reviewer's positive feedback and their view that the work is timely and highly relevant.
In the revised version of the manuscript and the replies below, we have addressed their points. In particular, we extended the treatment of the dressed-state description by adding a Supplementary Note (Note 1). We also designated another Supplementary Note (Note 4) to our correlation logic and an analysis of the associated error.

> **3.1. Reviewer 3:**
> The dressed states $|\alpha, N\rangle$ and $|\beta, N\rangle$ are introduced, but the physical meaning of α and β remains insufficiently specified. The authors argue that when the dressed-state splitting $\Omega_{\text{eff}}(t)$ matches the phonon spectral density, phonon emission leads to population inversion. However, the manuscript does not adequately discuss the theoretical modeling of the phonon spectral density, relying mainly on references to earlier work. It appears that by "matching," the authors refer to the frequency at which the phonon spectral density is maximal. Importantly, this condition can, in principle, be satisfied for both positive and negative laser detunings $\delta\omega_L$. Nevertheless, previous studies (e.g., Ref. [28]) have shown that phonon-assisted inversion depends strongly on the sign of the detuning. Furthermore, dressed-state populations themselves depend on the detuning sign due to asymmetric phonon emission and absorption processes. These aspects should be discussed more clearly.

Our reply:
We thank the Reviewer for the comment, which prompted us to add Supplementary Note 1, which provides a theoretical description of the dressed-state model. We also agree that our phrasing in the Main Text could have been more precise in some places and have implemented changes accordingly.
In particular, the Reviewer's understanding is correct that the effective Rabi frequency must be close to the frequencies that lead to strong QD-phonon interactions. As correctly pointed out, the form of the effective Rabi frequency

$$\Omega_{\text{eff}}(\text{t}) = \sqrt{\Omega^2(\text{t}) + \delta\omega_{\text{L}}^2} \quad (2)$$

is symmetric with respect to the sign of the laser-QD detuning $\delta\omega_L$ such that both red- and blue-detuned laser pulses may excite the QD. However, using a red-detuned laser requires the absorption (rather than emission) of a phonon, making this process much less efficient at cryogenic temperatures. Hence, these detunings are often excluded when describing phonon-assisted excitation, and we follow the same practice in this work by stating in the Main Text that we focus on $\delta\omega_L > 0$. Nonetheless, we acknowledge that the remark could have been more concise and, in the revised Main Text, we have included the rationale for focusing on $\delta\omega_L > 0$.
Furthermore, as also captured by the Reviewer, the strength of the LA phonon coupling may be characterized by the spectral phonon density, commonly denoted as $J(\omega)$. Although there are a few theoretical works deriving an analytical form for $J(\omega)$, they all share the same key features relevant to phonon-assisted excitation in general and re-excitation in particular. Namely, $J(\omega)$ vanishes for $\omega \to 0$ and beyond some cut-off frequency, and it has a "broad" peak that causes smooth changes in coupling strength when the dressed-state level splitting is tuned. The exact shape of $J(\omega)$ results from symmetry and geometric considerations of the QD (for example, see [Phys. Rev. B 78, 165311 (2008)]), but is not essential for the interpretation of our results. Hence, to avoid increasing the complexity of the manuscript more than necessary, we do not explicitly introduce these subtleties of $J(\omega)$ in the Main Text but mention them in the new Supplementary Note 1.

> **3.2. Reviewer 3:**
> The phonon-assisted excitation mechanism presented in the manuscript appears to operate primarily in the far off-resonant driving regime. This limitation should be explicitly stated and discussed, as it is important for assessing the general applicability of the proposed scheme.

Our reply:
The LA phonon-assisted scheme is indeed a non-resonant excitation method, and since it relies on the scattering of phonons, it is also incoherent. While these differences compared to resonant π-pulse excitation (RE) might pose limitations for some applications, they are highly beneficial for others, and there is a lively discussion about the ideal pumping scheme for each application.
For example, using RE, the laser light and QD emission must be separated using spatial filtering that is not compatible with widely-used confocal setups, or they employ polarization filtering that generally reduces the single-photon efficiency by 50 % (there are exceptions using charged excitons in specially tailored cavities). Using LA excitation, on the other hand, the laser is simply rejected using spectral filtering thanks to the large

laser-QD detuning. Furthermore, while coherence of the QD emission might be desirable in some cases, it must be avoided in others, including many cryptographic protocols (see Ref. 31 (33) [from here on, all references in brackets refer to the revised version and those without to the submitted version]). Another aspect is the practicality for real-world implementation. Using π-pulses to induce a Rabi rotation is sensitive to variation in the laser frequency or power, whereas the phonon-assisted excitation provides much more robustness thanks to the spectral width of the phonon coupling (see Ref. 29 (31)).
This debate has many more facets, and our Introduction can only brush them (lines 31-49), but we provide several references and mention key points, including that LA excitation is off-resonant and incoherent.
Prompted by this comment, we added the value of laser-QD detuning to the caption of Fig. 3.

3.3. Reviewer 3:

The authors state that the cavity influence is negligible in the reported experiments. While this may be reasonable, a more quantitative analysis or estimate (e.g., comparison of relevant rates or coupling strengths) would significantly strengthen this claim.

Our reply:
As stated in the Methods, the FWHM of the cavity mode is ≈1250 GHz while the maximum Stark-induced red-shift observed is ≈50 GHz. Furthermore, as shown in Fig. R1, which is a zoomed-in version of Fig. S1 (S2) and included here for the Reviewer's convenience, the cavity mode peak is slightly red-shifted with respect to the undisturbed QD emission frequency ω_0. As a result, any variation of the cavity's spectral response in the relevant regime (i.e., $\delta\omega/2\pi \in [0, -50]$GHz) is well below 1 % (almost limited by the measurement accuracy). In first approximation, the frequency dependence of the cavity-induced enhancement of the decay rate (Purcell enhancement) is directly proportional to the cavity's spectrum.

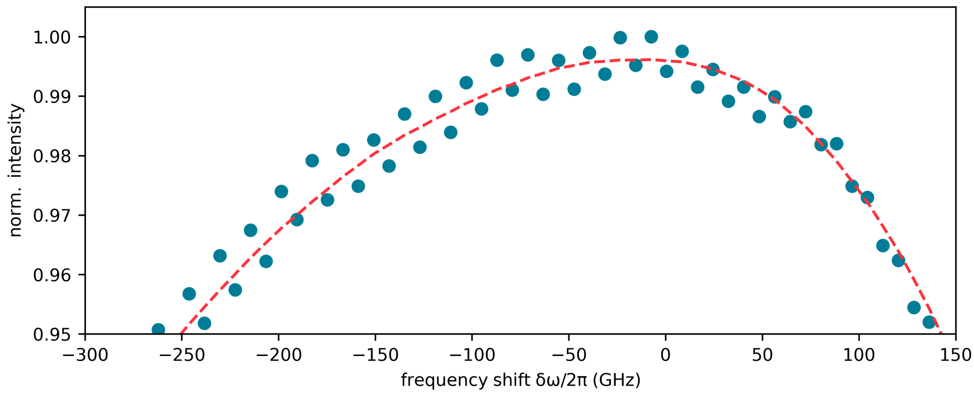

Review reply Figure 1: **Close-up of the cavity mode.** The cavity mode is measured using a strong above-band laser and collecting the emission of the sample on a spectrometer. The frequency is given as detuning from the spontaneous emission line, that is $\delta\omega = \omega - \omega_0$. The spectrometer resolution is ≈10 GHz per pixel. The dashed red line is obtained using a Savitzky-Golay smoothing filter.

In our opinion, this analysis justifies the assumption of negligible cavity influence. We

also believe that attempting to extract cavity-enhanced rates from simulations or experimental data could not further substantiate this claim due to the related uncertainties.

> **3.4. Reviewer 3:**
> The procedure used to define and separate the “first” and “second” photons through correlation gating requires clearer justification. In particular, a discussion of possible systematic biases introduced by this method, and how they may affect the extracted results, would improve the rigor of the analysis.

Our reply:
We agree with the Reviewer that when introducing a new measurement and analysis technique, it is best practice to provide a more detailed description and estimate of the associated errors. We therefore summarized the method and our error calculations in an additional Supplementary Note (Note 4).

> **3.5. Reviewer 3:**
> A brief discussion of how sensitive the re-excitation dynamics and phonon-assisted processes are to temperature would be valuable, especially for practical implementations.

Our reply:
We agree that the temperature dependence of an excitation process is of practical importance when using QDs as single-photon sources in real-world applications. Generally, elevated temperatures reduce the efficiency of phonon-assisted schemes using a blue-detuned laser. At low temperatures, the population is transferred efficiently from the upper dressed state to the lower dressed state, because emitting a phonon is much more likely than absorbing one (i.e., $|\alpha, N\rangle \rightarrow |\beta, N\rangle$ dominates over $|\beta, N\rangle \rightarrow |\alpha, N\rangle$). As the phonon occupation increases with higher temperatures, the asymmetry in transition rates lessens, approaching the point where the populations of the upper and lower dressed states tend to 0.5 (simulations vary but around 50 K to 80 K, see. Refs (6)-(7) of the Supplementary Information). When using a red-detuned laser, the excitation efficiency improves at higher temperatures but does not exceed 0.5, which remains too low to be of practical interest.
As the focus of this work lies on the re-excitation phenomenon and we do not anticipate any impact beyond the generally reduced efficiency of the population inversion, we believe that this discussion is more suitable for the Supplementary Information than the Main Text and have included it in Supplementary Note 1.

....

In addition to the Reviewers’ comments, an error in Fig. S8 was corrected in the revised version, where the sign of the laser-QD detuning in the legend was negative instead of positive. Also, in the meantime, references 6, 7, 21, 34, and 46 have been published in peer-reviewed journals, and the revised Main Text has been updated to cite those.
